# Supplementary material for: Mechanistic insights into PCBP1-driven unfolding of selected i-motif DNA at G1/S checkpoint
Source: Nat Commun. 2026 Feb 2;17:1149. doi: 10.1038/s41467-026-68822-5 (PMC12865031; doi:10.1038/s41467-026-68822-5)
Supplement: Supplementary file 1 — Supplementary Information [file 41467_2026_68822_MOESM1_ESM.pdf]

Supporting Information

Mechanistic Insights into PCBP1-driven Unfolding of Selected i-motif DNA at G_1/S checkpoint

Pallabi Sengupta[1], Natacha Gillet[2], Ikenna Obi[1], and Nasim Sabouri[1,3 #]

[1] Department of Medical Biochemistry and Biophysics, Umeå University, SE-901 87 Umeå, Sweden
[2] CNRS, ENS de Lyon, Université Claude Bernard Lyon 1, Laboratoire de Chimie UMR 5182, F-69342 Lyon, France
[3]Science for Life Laboratory, Umeå University, Umeå, Sweden

[#] Corresponding author: nasim.sabouri@umu.se
Phone: +46 786 50 00

Supplementary Methods

Electrophoretic mobility shift assay (EMSA) 2
Thermal-shift assay 2
Microscale Thermophoresis (MST) 3
Circular dichroism (CD) 3
Concentration-dependent CD melting assay 4
I-motif destabilization-trapping assay 4
Bromine footprinting 5
Isothermal titration calorimetry (ITC) 6
Purification of recombinant PCBP1 and KH mutants 6
High-resolution-primer extension assay 6
^{1}H 1D NMR 7
Docking and Molecular Dynamics (MD) simulation 7
Bioinformatic analyses 8
Cell culture and PCBP1-knockdown 8
qRT-PCR 9
Western blot 9
iMab, PCBP1, and BG4 Chromatin immunoprecipitation (ChIP) 10
Flow cytometry-based cell cycle analyses 10
Cell synchronization using double-thymidine block 11
Anti-BrdU dot-bot assay 11
Statistical analyses 11

Supplementary Tables

Supplementary Table 1: Oligonucleotides used in this study for *in vitro* studies 12
Supplementary Table 2: GC-richness of the PCR amplicons in iMab-ChIP-qPCR........ 12

Supplementary Table 3: Melting temperatures (T_m) of PCBP1 at different pH. 13
Supplementary Table 4: Apparent binding dissociation constants ($K_{D,app}$) calculated from MST binding curves 13
Supplementary Table 5: Melting temperatures (T_m) of KH1+2 and KH3 at different pH 13
Supplementary Table 6: Forward and reverse primer pairs used in ChIP-qPCR assays 14
Supplementary Table 7: Forward and reverse primer pairs used in qRT-PCR assays 14
Supplementary Figures 15
Supplementary References.. 32

Supplementary Methods:

Electrophoretic mobility shift assay (EMSA): For qualitative estimation of PCBP1's affinity towards folded and unfolded forms of i-motifs, we performed EMSA. 5 nM of the 5'-Cy5-labeled i-motif-forming sequences (iMfs), procured from Eurofins Genomics Europe (Supplementary Table S1) were incubated in assay buffers containing either 20 mM Bis-Tris (pH 6.4) (Zellbio B-020-250) or 20 mM Tris-HCl (pH 8.0) (Sigma T1503), supplemented with 100 mM NaCl and 0.25 mg/mL bovine serum albumin (BSA) (Merck A9647). Increasing concentrations of purified recombinant PCBP1 were then added to these mixtures. The reactions, prepared in a total volume of 50 µL, were incubated at 25 °C for 30 minutes to allow potential binding interactions. Following the incubation, 10 µL of 80% glycerol was mixed to each reaction tube and 20 µL of the reaction mixture was loaded onto 8% native PAGE (Polyacrylamide gel electrophoresis). The gel matrix was prepared with either 50 mM Bis-Tris (pH 6.4) or 50 mM Tris-HCl (pH 8.0), supplemented with 100 mM NaCl, to match the respective assay buffer conditions and pH during experiments. Electrophoresis was conducted at 70 V at 6 °C, using a 1× running buffer containing the same composition of either Bis-Tris or Tris-HCl buffer with NaCl as described. After electrophoresis, gels were imaged using an Amersham Typhoon scanner equipped with a Cy5 laser at 300 V with a resolution of 25 µm per pixel. Each assay was performed in triplicates.

Thermal-shift assay: pH tolerance of purified PCBP1 and KH-mutants (KH1+2 and KH3) was determined by thermal-shift assay using Sypro-orange-based protein thermal-shift dye kit (Applied biosystems; 4461146). We prepared 20 µL of 10 µM protein solutions in 20 mM MES buffer at pH 5.5, 6.0, 6.4 and in 20 mM Tris-Cl buffer at pH 7.0 and 8.0, supplemented with 100 mM NaCl into flat-bottom 96-well plates (BioRad) and heated in a CFX96TM realtime system (BioRad) from 25 ºC to 95 ºC in increments of 0.2 ºC. Changes in Sypro-orange fluorescence were monitored and the melting temperatures (T_m) (Supplementary Table S3, S5) were determined by calculating the derivative of the midpoint of the protein unfolding transition, defined by –dF/dT, representing the negative derivative of Sypro-Orange fluorescence signal with respect to temperature. Preparation of the proteins in the storage buffer (20 mM Tris-Cl (pH 8.0), 150 mM NaCl) was used as a reference. The experiment was performed in triplicates.

Microscale Thermophoresis (MST): We performed MST to study the interaction between PCBP1/KH mutants and 5′-Cy5-labelled oligonucleotides (iMfs and other control sequences including G4 and non-GC-rich sequences) by measuring the movement of oligos in response to a temperature gradient, allowing precise quantification of apparent binding affinities ($K_{D,app}$), in solution. 40 nM of 5'-Cy5-labeled iMfs were incubated in 20 mM MES (pH 6.4) or phosphate buffer (pH 7.0) or Tris-Cl (pH 8.0) with 100 mM NaCl and varying concentrations of PCBP1 or KH mutants (35 nM to 2.5 μM) in 20 μL reactions at 25 °C for 15 minutes. Then, samples were centrifuged at 11,000 × g for 10 minutes at 25 °C, followed by loading them into Monolith NT Capillaries (NanoTemper; SKU:MO-K022). Fluorescence intensities and MST were measured in Monolith NT.LabelFree (NanoTemper) instrument at LED excitation 30%. Data were normalized with MO.Control (v1.6.1) and fitted using 4PL equations that yields the apparent dissociation constants ($K_{D,app}$), where A denotes lower asymptote; B denotes upper asymptote. C is the inflection point or $K_{D,app}$ in nM).

D defines the Hill slope: $y(x) = A + \frac{B-A}{1+(\frac{x}{C})^D}$ .... (1)

Results were expressed as mean ± SD from triplicate measurements.

Circular dichroism (CD): To observe i-motif-spectral changes upon PCBP1/KH mutants' interactions, spectral analyses of PCBP1 and KH mutants, and pH dependent changes in PCBP1, CD spectra of 4 μM iMfs (procured from Eurofins Genomics Europe) or proteins were acquired using Jasco J1700-CD spectrophotometer. Oligos were first annealed into 20 mM MES (pH 6.4) and 100 mM NaCl. PCBP1 or KH-mutants were titrated into iMfs in increasing concentrations (1-4 μM) and incubated for 30 min at 25 °C before spectral acquisitions. To measure the transition pH (pH_T) of i-motifs, we acquired i-motif spectra at 4 μM concentration within a broad range of pH from 5.4 – 7.2[1]. CD scans were performed within 320-210/200 nm range and at 100 nm/min scanning speed. Data-points were obtained at 0.5 intervals and averaged over three accumulations. We used 1 mm-pathlength quartz cuvettes (Hellma) carrying 200 μL samples. Digital integration time and bandwidths were 2 s and 1 nm respectively. CD values were corrected for respective buffer contributions and converted to molar ellipticities (MCD) using the following formula (2), where θ is CD ellipticity in millidegrees, c is DNA concentration (mol/L), and l is path-length (cm):

$$\Delta\varepsilon(M^{-1} \cdot cm^{-1}) = \frac{\theta}{32980 \times c \times l} \text{........... (2)}$$

pH_T values were calculated by plotting fraction of folded i-motifs (calculated from MCDs at 287 nm (positive maxima)) vs pH, using the equation below:

$$f(folded) = \frac{MCD_i - MCD_{pH\ 7.2}}{MCD_{pH\ 5.4} - MCD_{pH\ 7.2}} \text{........... (3)}$$

We fitted these data-points using the Slogistic1 equations (1) in OriginPro 2020, where x_c = pH_T. Results were shown as means ± SD of triplicates.

For analysing the pH-dependent secondary structural changes in PCBP1, we uploaded the baseline-subtracted PCBP1-CD spectra at specific pH to the BeStSel web server[2] using default deconvolution parameters, and the 200–240 nm region was used for fitting. BestSel returned the fractional contents of secondary-structure classes (α-helix, parallel and anti-parallel β-sheet, and turn) as well as the fitted spectrum, RMSD (root mean square deviation) and NRMSD (normalised RMSD) goodness-of-fit values. RMSD defines the difference between the experimental and fitted spectra across the fitting wavelength range reflecting the absolute deviation in ellipticity units. NMRSD means the RMSD normalized to the experimental ellipticity range, giving a dimensionless measure of relative fit quality. For all the spectra in this study, RMSD and NRMSD values are within the recommended ranges.

Concentration-dependent CD melting assay: To assess whether the four i-motifs undergo intermolecular folding at 4 μM concentration under our experimental conditions (20 mM MES (pH 6.4) with 100 mM NaCl), we conducted concentration-dependent CD melting assays[3–6] across a concentration range of 2–24 μM. We examined the concentration-dependent melting of C6TC6 at our experimental conditions (pH 6.4) and within same buffer at pH 5.0, which served as a positive control, and had previously been studied as intermolecular i-motif[6]. CD melting experiments were conducted under identical instrumental parameters as described above. Spectra were recorded between 20 and 95 °C at 5 °C intervals with a heating rate of 1 °C/min. To ensure thermal equilibration, each temperature point was maintained for 180 s prior to acquisition. For measurements at 4 μM and higher concentrations, samples (200 μL) were analyzed in 1 mm pathlength quartz cuvettes (Hellma), whereas the 2 μM samples (2 mL) were measured using 10 mm pathlength quartz cuvettes. CD signals at the positive ellipticity maximum (~287 nm) were normalized by calculating fraction of folded using the following relation:

$$\text{Fraction of folded} = \frac{(\text{ellipticity}_i - \text{ellipticity}_{95\,°\text{C}})}{(\text{ellipticity}_{20\,°\text{C}} - \text{ellipticity}_{95\,°\text{C}})} \dots (4)$$

Then melting curves were generated by plotting the fraction of folded values versus temperature range; and fitting the datapoints using 4PL equations that yields the melting temperatures (T_m), where A denotes lower asymptote (minimum fraction folded, *e.g.*, fully unfolded → ≈0); B denotes upper asymptote (maximum fraction folded, *e.g.*, fully folded → ≈1). C is the inflection point or T_m in °C). D defines the Hill slope: $y(x) = A + \frac{B-A}{1+(\frac{x}{C})^D}$ .... (1)

I-motif-destabilization trapping assay: To estimate the i-motif unfolding kinetics by PCBP1 and KH mutants, we optimized an i-motif- destabilization trapping assay. In this assay, 5′-Cy5-labelled iMfs

substrates were prepared at 5 nM in 20 mM MES (pH 6.4), 50 mM NaCl, 2 mM $MgCl_2$, and 0.25 mg/mL BSA followed by incubation for 30 min at 25 °C in presence of 5 nM PCBP1 or KH mutants. Reactions were performed by adding 10 nM i-motif-trap-complementary oligos (Supplementary Table S1), specific for each iMfs at 25 °C up to 20 minutes. At indicated time-points, 10 µL of reaction mix was quenched with 1:1 stop buffer (40% glycerol, 60 mM EDTA, 0.6% SDS, 0.5 µM unlabelled iMfs) and analyzed by 20% native PAGE at 110 V. Gels were imaged using Amersham Typhoon scanner with a Cy5 laser at 300 V with a resolution of 25 µm per pixel and quantified with ImageJ. Negative controls lacked i-motif-trap-complementary oligos, while in positive controls, iMfs were incubated with i-motif-trap-complementary oligos for 24 hours at room temperature to allow complete hybridization. The fraction of hybridization between unfolded i-motif and their complementary trap oligos was estimated and normalized by dividing the intensity of trapped or hybridized products with the sum of trapped and unhybridized band intensity. Data-points plotted against time-points were fitted using a single-exponential function equation (4) to calculate the rate of i-motif-unfolding (k), where $F_{max}(t)$ denotes the fractional maximal value (or normalized signal) at time t. Experiments were repeated three times.

$$\mathrm{F}_{\max}(t) = 1 - e^{-kt} \text{.... (5)}$$

Bromine footprinting: To map the destabilized $C:CH^+$ pairs in *cMYC*-i-motif upon PCBP1 or KH mutant binding, we performed bromine footprinting assay. This assay relies on preferential bromination of single-stranded or unpaired Cs rather than those engaged in $C:CH^+$ base pairs due to the chemical accessibility and electronic environment of Cs in i-motif structure. Bromine reacts with Cs via electrophilic substitution at the C5 position in the pyrimidine ring. In single-stranded or unpaired Cs, the C5 position is more exposed and readily accessible to bromine while in intercalated $C:CH^+$ base pairs formed within i-motifs, Cs are stacked and hydrogen-bonded, reducing their accessibility. Further, protonated Cs in i-motif at N3 position has altered electron density that affects the electrophilic susceptibility of the C5 position, making it less reactive toward bromination[7]. 10 µM of 3′-Cy5-labeled *cMYC*-iMfs were annealed in 20 mM MES (pH 6.4) or 20 mM phosphate buffer (pH 7.0) with 50 mM NaCl by heating at 95 °C for 5 minutes, followed by slow cooling overnight. 5 µM iMfs pre-incubated with or without 5 µM PCBP1/KH-mutants for 30 minutes at 25 °C was treated with molecular bromine formed *in situ* by mixing equimolar KBr (Sigma Aldrich; 221864) and $KHSO_5$ (Sigma Aldrich; 228036) (20 µM) for 20 minutes at room temperature. Reactions were stopped by 80 µL of 0.3 M sodium acetate (pH 7.0) and 25 µg/mL calf thymus DNA on ice. Unreacted bromine was removed by two successive ethanol precipitation. The DNA pellet was resuspended in 100 µL of 100 mM piperidine (Sigma; 104094), heated at 90 °C for 20 minutes to induce bromination-specific strand cleavage, then washed thrice and dried using speed-vac. Samples were dissolved in 95% formamide (VWR; A2156) and 20 mM EDTA, heated at 95°C for 5 minutes, and snap-cooled. Purine- and pyrimidine-specific reactions were performed using 4% formic acid (Sigma Aldrich; 5438040100) and hydrazine (Sigma Aldrich;

309400) to generate A+G and C+T sequencing markers respectively following Maxam-Gilbert reactions[8]. Samples were analyzed on 20% denaturing PAGE at 60 W, imaged using Amersham Typhoon scanner with Cy5 laser (500 V, 25 μm pixel resolution), and quantified with ImageJ. Each assay was performed in triplicates.

Isothermal titration calorimetry (ITC): Thermodynamic binding profiles between iMfs and proteins (PCBP1 and KH-mutants) were determined using ITC with a MicroCal Auto iTC200 at 25 °C at both pH 8.0 (20 mM Tris-HCl + 150 mM NaCl) and pH 6.4. For the binding studies involving KH1+2 and KH3, 5 μM of KH1+2 was prepared in 20 mM MES (pH 6.4) and 150 mM NaCl and placed in the calorimetric cell. The syringe contained 350 μM KH3 in the same buffer. To investigate the interactions between KH-mutants (KH1+2 and KH3) and i-motif, 5 μM KH1+2 was pre-incubated with an equimolar concentration of *cMYC*-i-motifs for 30 minutes before being placed in the cell, while the syringe was filled with 350 μM KH3. For binding studies between proteins and iMfs, 5 μM of each protein was loaded into the cell, and 300 μM of iMfs was placed in the syringe. Control experiments were conducted simultaneously by injecting the same concentrations of substrates into a buffer without ligand to account for the heat of dilution. Oligonucleotides were injected 20 times at 150-second intervals into the calorimeter cell to achieve binding saturation. Data analysis was performed using the Malvern Microcal PEAQ-ITC software, employing a 'one-site' binding model to obtain the best-fit values for number of binding sites (N), changes in enthalpy (ΔH), entropy (ΔS), free energy (ΔG) of binding reactions.

Purification of recombinant PCBP1 and KH mutants: cDNAs of PCBP1, KH1+2, and KH3 were cloned into the pET-His1a vector for overexpression of recombinant PCBP1 in *Escherichia coli* BL21(DE3) cells. PCBP1 was induced at 18 °C before harvesting by centrifugation. The cell pellet was resuspended in lysis buffer (50 mM NaP 8.0, 500 mM NaCl, 10% glycerol, 0.2% Triton X100, 10 mM Imidazole, 5 mM β-mercaptoethanol, DNase) followed by sonication. The sample was centrifuged (30 min, 20,000 × g, 4 °C), and the supernatant was incubated (1.5 h, 4 °C) with ThermoS NiNTA previously equilibrated in lysis buffer. The mixture was then poured into a gravity flow column and the resin was washed with wash buffer (wash buffer 1: 50 mM NaP pH 8.0, 500 mM NaCl, 10% glycerol, 0.2% Triton X100, 10 mM Imidazole, 5 mM β-me; wash buffer 2: 50 mM NaP pH 8.0, 1 M NaCl, 10% glycerol, 0.2% Triton X100, 10 mM Imidazole, 5 mM β-me). The elution was performed stepwise with two elution buffers (Elution buffer 1: 50 mM NaP pH 8.0, 500 mM NaCl, 20 mM Imidazole, 5 mM β-me; elution buffer 2: 50 mM NaP pH 8.0, 300 mM NaCl, 300 mM Imidazole, 5 mM β-me). The eluates were analyzed by SDS-PAGE, pooled together according to their purity, and desalted by dialysis in 20 mM NaP pH 8.0, 150 mM NaCl, 5 mM β-me. His tag was cleaved by TEV protease and final dialysis was done in 20 mM Tris-Cl 8.0, 150 mM NaCl, 20% Glycerol, 1 mM DTT (Dithiothreitol).

High-resolution-primer extension assay: To examine the effect of i-motif/hairpin formation to slow down *in vitro* replication, we performed primer extension assays[9]. 5′-TET (Tetrachlorofluorescein)-labelled primer (1 μM) was annealed to i-motif-forming templates (1.5 μM) in 75 mM NaCl and 6 mM $MgCl_2$ by heating at 95 °C for 5 min followed by slow-cooling overnight. 40 nM of annealed samples were incubated in 50 μl reactions containing 20 mM MES (pH 6.0), 6 mM $MgCl_2$, 0.2 mg/ml BSA, and 0.05 μU/L Klenow fragment (Thermo Scientific; EP0051) with/without 40 nM PCBP1 or KH-mutants for 20 minutes at 25 ºC. Primer extension was started with 0.2 mM dNTPs for and continued for specific time-points. Reacted samples were collected at designated time-intervals, quenched with stop solution (95% formamide, 20 mM EDTA), denatured at 95°C for 5 minutes, and snap-cooled. 5 μl was loaded on 12% denaturing PAGE (8 M urea (VWR; 28877.292), 25% formamide, 1×TBE), and run at 60 W in 1× TBE running buffer. The gel was visualized using Amersham Typhoon (GE Healthcare) with Cy3 laser (500 V, 25 μm pixel resolution) and quantified using ImageJ. Each experiment was performed in triplicates.

^{1}H 1D NMR: NMR experiments were conducted using a Bruker DRX 850 MHz NMR spectrometer, equipped with a 0.7 mm ultra-fast MAS probe to understand the impact of PCBP1/KH mutants binding on i-motif's structural dynamics. For the experiments, 100 μM of iMfs from *cMYC*, *BCL2* (wild-type and mutants), hTeloC, and *ILPR* were prepared and annealed in 20 mM MES buffer (pH 6.4) containing 100 mM NaCl in a solvent mixture of 90% water and 10% D_2O. The 1D ^{1}H NMR experiments were performed in 3 mm NMR tubes (Bruker; Z172598) with an active sample volume of 200 μL. Spectral referencing was performed using an internal standard, TSP [3-(trimethylsilyl)-2,2′,3,3′-tetradeuteropropionic acid] (Sigma; 450510), set at 0.0 ppm. In the 1D proton spectra, imino proton resonances corresponding to $C:CH^+$ bonds in the i-motifs were observed at 15-16 ppm, while resonances indicative of hairpins involving Watson-Crick base pairs were detected at 12-14 ppm. These measurements were obtained using the standard Bruker pulse program "zgesgp" with a spectral width of 20 ppm, 256 scans (ns), acquisition time 2 s, and a calibrated pulse length (pl) of 9.48 μs. NMR titrations were performed by incrementally adding aliquots of purified proteins (PCBP1 and KH-mutants) to the 100 μM iMfs. Samples were thoroughly mixed and allowed to reach thermal equilibrium. Proton spectra were recorded at each titration point, following an incubation period of 15 minutes at 298 K. Data acquisition and processing were carried out using Topspin 4.2.0 (Bruker).

Docking and MD simulations: Molecular Dynamics simulations (MDs) were performed using Amber20 package on structure obtained from Alphafold[10] and RoseTTAFold[11] or RoseTTAFoldNA[12]. Amberff14SB[13] and parmbsc1[14] force fields were used to model protein and DNA i-motif respectively, with the CUFIX correction for ionic interaction[15] and Amberff14IDPSFF[16] force field for the intrinsically disordered part of PCBP1 between domains 2 and 3 (residues 170 to 268). The protonation state of amino acids of PCBP1 protein was determined on the basis of PropKa[17] calculations. All the

systems were solvated in a cubic box of TIP3P water with a solvent buffer of at least 12 Å and about 0.10 M of NaCl salt taking into account the neutralization of the box. Periodic conditions were applied in combination with Particle Mesh Ewald method for electrostatic and a cutoff of 10 Å for intermolecular interactions. All the systems were first minimized for 10,000 steps (5,000 steepest descent, 5,000 conjugated gradient) and then heat from 0 to 300 K during 30 ps in NVT ensemble with a timestep of 1 fs. Equilibration (100 ns) and production (1 to 3 µs) were run in NPT ensemble at 300 K and 1 bar using Langevin thermostat. SHAKE algorithm was applied to constrain hydrogen-heavy atoms bonds and maintain a time step of 2 fs. Trajectories were analysed using CPPTRAJ[18]. First, MD simulations were performed on 6 conformations of PCBP1 alone, one from AlphaFold prediction, 5 from RoseTTAFold prediction. We selected the representative structure of the most important cluster from a cluster analysis based on the RMSD of the protein residues and hierachical agglomerative approach for each trajectory of 100 ns. These structures were combined with the 8AYG[19] structure for i-motif DNA with the ILPR sequence using RoseTTAFoldNA docking. Five docked conformations (Fig. S21A) were selected based on the localization of the DNA towards the different domains, especially KH1 and KH3, and DNA-RNA binding part of these domains. Three simulations setups were created with only neutral cytosines in the i-motif, one neutral (set a); one with one positively charged cytosine for on half on the cytosine pair (set b); and one with one positively charged cytosine per i-motif cytosine pair (set c). The production runs last 1 µs for set a structures 1, 4 and 5; 2 µs for set a structures 2 and 3 and for all structures of set c; 3 µs for all structures of set b. To compare the structural features of the KH folds between hnRNP-K and PCBP1, we aligned them using VMD software (https://www.ks.uiuc.edu/Research/vmd/)[20].

Bioinformatic analyses: *De novo* motif discovery was performed on the bed file extracted from the ChIP-seq datasets using MEME-ChIP web-interface setting the following parameters. Enrichment mode: Classic; Set of known motifs: Eukaryote DNA, Human and Mouse (HOCOMOCO v12 CORE), background model: 1st order model, MEME Site Distribution: 0 or 1 occurrence, MEME motif count: 3 and MEME Motif width: 6–15 wide. We used plotheatmap tool in Galaxy to visualize ChIP-enrichment values around 2000 bp around the TSS (transcription start site). To compare functional overlap and uniqueness between PCBP1 and other hnRNP family proteins, we used previously published i-motif interactome proteomics data[21] that identifies candidate i-motif–binding proteins. From this dataset, all proteins annotated to the hnRNP family were extracted for gene ontology (GO) enrichment of Biological Process (BP) terms using the PANTHER Classification System, applying Fisher's exact test with FDR correction. Enriched GO terms were visualized using a Sankey diagram generated in RStudio 2024.09.0+375 and R 4.4.0.

Cell culture and PCBP1-knockdown (PCBP1-KD): HeLa cells were cultured at required density in Dulbecco's modified Eagle's medium (DMEM) (Gibco; 10565018) in presence of 10% FBS (Fetal

bovine serum) (Gibco; A5256701) at 37 °C and 5% CO_2 in the incubator. PCBP1 knockdown (KD) was performed using small interfering RNA (siRNA) (PCBP1 Human siRNA Oligo Duplex (Locus ID 5093, Origene; SR303372)) transfection with Lipofectamine 3000 (Thermo Fisher Scientific; L3000008) according to the manufacturer's protocol. Briefly, cells were seeded in 6-well plate at a density of 10^6 cells per well and allowed to adhere overnight. The following day, 5 ng of siRNA targeting PCBP1 (siPCBP1) or 5 ng of non-targeting scrambled siRNA (siScramble, negative control) was diluted separately in Opti-MEM reduced serum medium (Thermo Fisher Scientific; 31985070). In parallel, Lipofectamine 3000 reagent was also diluted in Opti-MEM and incubated for 5 minutes at room temperature. The diluted siRNA was then combined with the diluted Lipofectamine 3000 reagent, mixed gently, and incubated for 10–15 minutes at room temperature to allow complex formation. The siRNA-lipid complexes were added dropwise to the cells, and the plates were gently swirled to ensure even distribution. Cells were then incubated at 37°C with 5% CO_2 for 48 hours, and the KD was confirmed by western blot analyses with anti-PCBP1 antibody.

qRT-PCR: To inspect the transcript levels of *c-MYC*, *BCL-2*, and *ILPR* genes upon PCBP1-KD, we performed quantitative real time PCR. HeLa cells are sub-cultured into 6-well microtiter plates at a density of 1×10^6 cells per well and treated by PCBP1-KD as described above. Total RNA is isolated from both untreated and KD cells using Qiagen RNeasy Mini Kit (Qiagen; 74104) as per manufacturer's instructions. cDNA was prepared using UltraScript 2.0 cDNA synthesis kit (PCR Biosystems; PB30.31-02) by incubating a 20 μL reaction containing 2 μg RNA, cDNA synthesis mix and Ultrascript 2.0 reverse transcriptase at 50 °C for 30 min followed by a denaturation 95 °C. qRT-PCR reactions contain 1x SyGreen mix (PCR Biosystems), 0.5 μM forward and reverse primers and cDNA. The PCR program was 95 °C for 3 min (1 cycle) followed by a three-step reaction of 95 °C for 10 s (denaturation), 57 – 60 °C for 25 s (annealing), and 72 °C for 20 s (elongation) (35 cycles) and performed in CFX Real Time System (C1000 Thermal cycler (BioRad)). Housekeeping gene, *GAPDH* is used as an internal control to normalize the variability in target mRNA expression levels and *HTR6* transcript levels are shown as negative control. qRT-PCR primers (Supplementary Table S7) are designed using Primer-BLAST, NCBI, and analyzed in OligoAnalyser 3.1-IDT.

Western blot: To confirm PCBP1-KD and estimate DNA double-strand breaks upon PCBP1-KD, we performed western blot analyses of PCBP1 and γH2AX (H2AX histones phosphorylated at Ser139) respectively in HeLa cells upon 48 hours of PCBP1-KD. Cells were seeded at a density of 7×10^5 cells per well into 60 mm dishes and allowed to attach overnight. At 48 hours post-transfection, total protein was extracted from the cells using freshly prepared RIPA lysis buffer (50 mM Tris (pH 8.0), 150 mM NaCl, 1% NP-40, 0.1% SDS, 0.5% sodium deoxycholate) supplemented with Phenylmethylsulfonyl fluoride (PMSF) and protease inhibitor (complete mini EDTA-free tablets, Roche; 5892791001). Protein concentration was quantified using the BCA assay (Pierce™ BCA Protein Assay Kit; 23225).

30 μg protein lysates were resolved on mini protean TGX precast SDS-PAGE gels (BioRad) and transferred onto nitrocellulose membranes (Amersham™ Protran® Premium Western blotting membranes). Membranes were blocked with 5% non-fat milk in 1× Tris-buffered saline (TBS) with Tween 20 (Amresco; SKU QBIC20726) for 1 hour at 4°C. Then, membranes were briefly washed with 1× TBST and incubated overnight at 4°C with rabbit monoclonal anti-PCBP1 primary antibody (dilution 1:1000, Abcam; clone number: EPR11049(B), catalog number: ab168377, IgG isotype; Lot number: 1050664-5), or rabbit phosphor-histone H2AX (Ser 139) antibody (dilution 1:1000, Cell signalling technologies; Catalog number 2577S; clonality polyclonal; Isotype: IgG; lot number 14) and mouse monoclonal anti-β-Actin antibody (dilution 1:5000, Abcam; catalog number: ab8224; isotype: IgG1; Lot number: 1051636-14). Then, membranes were incubated with HRP-conjugated Goat anti-mouse/anti-rabbit polyclonal IgG (H+L), (dilution 1:5000, Thermo-fisher Scientific; catalog number: 31460) for 2 hours at room temperature. Target proteins were visualized on membranes using Western Supersignal West pico chemiluminescence substrate (Thermo-fisher) and images are captured using ChemiDoc MP system with Image Lab™ software. After incubation with each antibody, membranes were washed for 15 min at room temperature three times with 1× TBST. β-Actin was used as a loading control.

iMab, PCBP1, and BG4 Chromatin immunoprecipitation (ChIP): 2×10^6 HeLa cells were crosslinked in 1% formaldehyde (Thermo-Fisher; 28908) for 10 min at room temperature followed by quenching with 125 mM glycine. After quenching, cells were washed with ice-cold 1× PBS and collected by centrifugation at 500 × g for 5 minutes at 4 °C. Cells were lysed on ice in ChIP lysis buffer (50 mM HEPES, pH 7.4; 140 mM NaCl; 1 mM EDTA; 1% Triton X-100; 0.1% Na-deoxycholate) for 10 min at 4°C. Chromatin was isolated and sheared to 200-600 bp using the Covaris E220 system. After RNase A treatment (10 μg/mL) (Thermo-fisher scientific; EN0531) at 37°C for 30 minutes, chromatin was incubated overnight (4 °C) with 2 μg iMab antibody (Absolute Antibody) or 500 ng BG4 antibody (in-house prepared)[22] or rabbit monoclonal anti-PCBP1 primary antibody (1:500, Abcam) in ChIP lysis buffer containing 1% non-fat milk in 200 μL, shaking at 25 ×g. For iMab and BG4, DYKDDDDK Tag (D6W5B) Rabbit mAb anti-FLAG antibody (Cell Signalling, catalog number: 14793S, Clonality: Monoclonal; IgG isotype; Lot number: 7) (8 μg) bound to 10 μL dynabeads protein-G (Invitrogen; 10003D) was prepped by shaking at 130 ×g for 2-4 hours at 4 °C, then washed and incubated with 80 μL chromatin-iMab complex at 33 ×g for 4 hours at 4 °C. Beads were washed four times with 100 μL ChIP lysis buffer and twice with 100 μL wash buffer at 4 °C, then immunoprecipitants were eluted with TE buffer (10 mM Tris-Cl, pH 8.0; 1 mM EDTA) containing 0.5 mg/mL Proteinase K by incubation at 37 °C for 1 hour and 65 °C for 2 hours. Eluates were purified using the ChIP-DNA Clean and Concentrate kit (ZYMO Research; D5201) following manufacturer's protocol. qPCR was conducted using primers (Supplementary Table S6) targeting promoters with iMfs or non-i-motif-forming control regions. ChIP enrichment was quantified using the percentage of input method, where input DNA

corresponds to a fraction of total chromatin collected before immunoprecipitation. Rabbit anti-FLAG IgG was used as the mock control for iMab and BG4, and anti-rabbit IgG was used for anti-PCBP1 ChIP. For each sample, the percentage of input was first calculated for both target loci and negative control regions. Fold enrichment of target loci was then determined by dividing the percentage of input of the target by the corresponding negative control. This two-step calculation allowed us to account for both IP efficiency and background signal, ensuring that the observed enrichment was specific to i-motif-forming regions.

Flow cytometry-based cell cycle analyses: HeLa cells were sub-cultured at a density of 1×10^6 cells per well. Following treatment with either double-thymidine block or PCBP1-KD as described above, cells were harvested using trypsinization and subsequently washed with cold 1× PBS. The cells were then fixed with chilled 70% ethanol and stored at −20 °C overnight. After fixation, the cells were centrifuged at 850 × g for 15 minutes at 4 °C. The resulting pellets were resuspended in 500 µL of FxCycle PI/RNase staining solution (Invitrogen; F10797) and incubated in the dark at 4 °C with gentle rocking for 4 hours. DNA content was analyzed using BD CSampler Plus Software version:1.0.34.1. Gating of single cells, histograms of DNA content (Propidium iodide (PI) fluorescence intensity) were analyzed in FCS Express. The percentage of cells in each phase of the cell cycle (G_1, S, and G_2/M) was determined by fitting the data into Cox multivariate analyses.

Cell synchronization using double-thymidine block: HeLa cells were synchronized at the G_1/S boundary using a double-thymidine block protocol. Cells were seeded at an appropriate density (30–40% confluence) in DMEM supplemented with 10% fetal bovine serum (FBS) and allowed to adhere overnight. The following day, thymidine (2 mM final concentration) (Sigma Aldrich; 89270) was added to the culture medium and incubated for 18 hours to inhibit DNA synthesis by depleting the nucleotide pool. Cells were then washed three times with 1× PBS and released into fresh, thymidine-free medium for 9 hours to allow progression through the cell cycle. Subsequently, a second thymidine treatment (2 mM final concentration) was applied for another 15–18 hours to achieve synchronization at the G_1/S transition. After the second block, cells were either harvested at the G_1/S boundary after 2 hours or released into fresh medium for 4 hours to obtain synchronized populations in S phase. Synchronization efficiency was confirmed by flow cytometry analysis of DNA content as described above.

Anti-BrdU dot-bot assay: HeLa cells were synchronized at G_1/S boundary using double-thymidine block method as described above. After second thymidine block, cells were released into fresh medium at four time-points at an interval of 1 hour up to 4 hours. A total of 1.3 µg genomic DNA isolated from cells using at each time-point using DNeasy Blood & Tissue Kit (Qiagen; 69504), and were heated at 95 °C and immediately cooled on ice to denature the DNA. 0.3 M final concentration of NaOH was added, and DNA was loaded onto a Hybond-N+ membrane (GE Healthcare) using a Bio-Dot Microfiltration Apparatus (Bio-Rad). The membrane was blocked overnight with 1% non-fat milk in

Phosphate Buffered Saline (PBS) and later incubated with Rat anti BrdU BU1/75 (ICR1) (Abcam; catalog number: ab6326, Clonality: monoclonal, subtype: IgG2a;) (1:1000 dilution) in 1% non-fat milk. After washing the membrane for 15 min twice with PBS, it was incubated with goat anti-rat IgG antibody peroxidase (Merck; catalogue number: DC01L-200UG) (1:5000 dilution) in 1% non-fat milk, and dots were developed using a chemiluminescent reagent (Western Supersignal West pico chemiluminescence substrate (Thermo-fisher; 34580)) and images are captured using ChemiDoc MP system with Image Lab™ software.

Statistical analyses: All statistical analyses in this study were performed using GraphPad Prism or OriginPro 2020. One-way ANOVA followed by Tukey-Kramer post hoc test was used for ChIP enrichment analyses to compare multiple groups. For all other experiments (*e.g.*, comparing $K_{D,app}$ values between PCBP1/KH mutants and multiple i-motifs), a two-tailed Student's *t*-test was used to assess statistical significance. Data from biochemical and cellular studies are presented as mean ± standard deviation (SD) from three biological replicates. A p-value < 0.05 was considered statistically significant.

Supplementary Tables:

Supplementary Table 1: Oligonucleotides used in this study for in vitro studies. All oligonucleotides are procured from Eurofins Genomics Europe in lyophilised form.

Name	Sequence (5´-3´)	Experiments
cMYC-iMfs	AATCCCACCCCTCCCATCCCTT	CD, EMSA‡, MST‡,
BCL2-iMfs	CAGCCCCGCTCCCGCCCCCTTCCTCCCGCGCCCGCCCCT	
ILPR-iMfs	TGTCCCCACACCCCTGTCCCCACACCCCTGT	
hTeloC-iMfs	CCCTAACCCTAACCCTAACCCT	
C7T3-iMfs	CCCCCCCTTTCCCCCCCTTTCCCCCCCTTTCCCCCCC	
Non-GC	AATTGATGTTATTCTTCTTATT	
cMYC-G4	AAGGGATGGGAGGGGTGGGATT	
mutC	TACACTCACACTCACACTCACACTCAA	
C-RNA	ACCCCGCCCCUCCCUAUUCCCGUCCCU	
BCL2-G10TG12T	CAGCCCCGCTCCCGCCCCCTTCCTCCCTCTCCCGCCCCT	CD
BCL2-G_{all}→T	CATCCCCTCTCCCTCCCCCTTCCTCCCTCTCCCTCCCCT	
C6TC6	CCCCCCTCCCCCC	
cMYC-iMfs-Br*	AATCCCACCCCTCCCATCCCTTTTT-Cy5	Bromine footprinting
cMYC-iMfs-PE	AATCCCACCCCTCCCATCCCTTatatatatatCGGACGCTCGACGC CATTAATAATGTTTTCA	High resolution primer
BCL2-iMfs-PE	CAGCCCCGCTCCCGCCCCCTTCCTCCCGCGCCCGCCCCTatata tatatCGGACGCTCGACGCCATTAATAATGTTTTCA	
Non-i-motif-PE	GAGACCATTCAAAAGGATAATGTTTGTCATTatatatatatCGGAC GCTCGACGCCATTAATAATGTTTTCA	

BCL2-m1-PE	CAGCCCCGCTCCCGCCCCCTTCCTCCCTCTCCCGCCCCTatatat atatCGGACGCTCGACGCCATTAATAATGTTTTCA	extension assay
BCL2-m3-PE	CATCCCCTCTCCCTCCCCCTTCCTCCCTCTCCCTCCCCTatatata tatCGGACGCTCGACGCCATTAATAATGTTTTCA	
TET-primer†	(TET)-TGAAAACATTATTAATGGCGTCGAGCGTCCG	
cMYC-trap	AAGGGATGGGAGGGGTGGGATT	I-motif destabilization -trapping assay
BCL2-trap	AGGGGCGGGCGCGGGAGGAAGGGGGCGGGAGCGGGGCTG	
ILPR-trap	ACAGGGGTGTGGGGACAGGGGTGTGGGGACA	
hTeloC-trap	AGGGTTAGGGTTAGGGTTAGGG	

‡ In these experiments, oligonucleotides are all Cy5 (cyanine 5) labelled at 5´-ends.
**cMYC-iMfs-Br* is Cy5 labelled with T_3-linker at 3´-end.
† TET primer is Tetrachlorofluorescein (TET)-labelled at 5´-end.

Supplementary Table 2: GC-richness of the PCR amplicons in iMab-ChIP-qPCR**:**

	**GC-richness (%)**
cMYC	56.6
BCL2	75.1
ILPR	67.9
PDGFa	81.2
VEGFa	73.7
HIF1a	72.1
ARHGEF10L	51.2
GAPDH	54.1
HTR6	56.9
IL36G	49.2

Supplementary Table 3: Melting temperatures (T_m) of PCBP1 at different pH, obtained from thermal shift assay. Errors calculated from 5 biological replicates (n = 5)

pH	Melting temperatures (T_m) (ºC)
8.0	55.1 ± 3
7.0	54.9 ± 2.5
6.4	54.5 ± 2.1
6.0	50.5 ± 1.6
5.5	48.6 ± 1.2

Supplementary Table 4: Apparent binding dissociation constants ($K_{D,app}$) calculated from MST binding curves showing binding profiles between PCBP1 and iMfs or control sequences at pH 6.4, 7.0, and 8.0. Errors calculated from 3 biological replicates (n = 3)

Apparent binding dissociation constants ($K_{D,app}$) (in nM)
MST

		pH 6.4	pH 7.0	pH 8.0
iMfs	cMYC	238 ± 5.4	982 ± 19.5	978 ± 21.8
	ILPR	255 ± 9.5	764 ± 13.7	757 ± 10.5
	BCL2	217 ± 6.6	972 ± 7.8	978 ± 8.5
	hTeloC	283 ± 10.6	745 ± 6.6	748 ± 8.8
	C7T3	189 ± 3.4	191 ± 2.4	288 ± 10.8
control	cMYC-G4	> 3000	2892 ± 24.9	2994 ± 26.2
	Non-GC	1955 ± 23.4	2032 ± 33.6	1525 ± 13.4

Supplementary Table 5: Melting temperatures (T_m) of KH1+2 and KH3 at different pH, obtained from thermal shift assay. Errors calculated from 5 biological replicates (n = 3)

pH	Melting temperatures (T_m) (ºC)	
	KH1+2	KH3
8.0	55.1 ± 1.3	55.6 ± 2.3
7.0	54.5 ± 1.6	55.2 ± 1.8
6.4	54.1 ± 2.3	55.1 ± 1.2
6.0	51.2 ± 0.23	54.7 ± 1.1
5.5	50.2 ± 0.22	54.4 ± 1.7

Supplementary Table 6: Forward and reverse primer pairs used in ChIP-qPCR assays:

**Primers**	**Primer sequences (5′-3′)**
PDGFa-F	GGAGAGGGTTATAGCGCCG
PDGFa-R	ACGAACCCCGAGCGCTTC
cMYC-F	AGGCGCGCGTAGTTAATTCA
cMYC-R	CATTATAAAGGGCCGGTGGG
BCL2-F	TGTGACGTTACGCACAGGAA
BCL2-R	TTCTCCTCCTCCTGGTCCTG
Hif1a-F	CTCTTTCCTCCGCCGCTAAA
Hif1a-R	CCCAGACCCGCTCTCCAG
VEGFa-F	ATCCTCCTGTCCCCTCAGAC
VEGFa-R	CCGCTACCAGCCGACTTTTA
HTR6-F	GGCGATTTGTCCAATATTTCCC
HTR6-R	CTGTGACCTGCCCTTATCC
ARHGEF10L-F	TGCCAAGTTACTCTCAGTTCTG
ARHGEF10L-R	AGCCAAACCTCCAAGAACAA
IL36G-F	GCCCACCTCTTTACTTCCTTA
IL36G-F	AACACTCTTTCAGCTCCATCC
ILPR-F	CTTTCCACATTAGACCAGGAG
ILPR-R	GACAGGGGTCCGGGGGACAG
GAPDH-F	TCCAATTCCCCATCTCAGTC
GAPDH-R	TAGTAGCCGGGCCCTACTTT

Supplementary Table 7: Forward and reverse primer pairs used in qRT-PCR assays:

Primers	Primer sequences (5′-3′)
cMYC-F	CCTGGTGCTCCATGAGGAGAC
cMYC-R	CAGACTCTGACCTTTTGCCAGG
BCL2-F	ATCGCCCTGTGGATGACTGAGT
BCL2-R	GCCAGGAGAAATCAAACAGAGGC
cMYC-P1-F	CTTGGCGGGAAAAAGAACGG
cMYC-P1-R	AGTTAGATAAAGCCCCGAAAACC
BCL2-P1-F	ATGTGTGTGGAGAGCGTCAACC
BCL2-P1-R	TGAGCAGAGTCTTCAGAGACAGCC
HTR6-F	GCATCCTGCTGGGCATGTTCTT
HTR6-R	CCATGTGAGGACATCGAAGAGG
ILPR-F	ACGAGGCTTCTTCTACACACCC
ILPR-R	TCCACAATGCCACGCTTCTGCA
GAPDH-F	GTCTCCTCTGACTTCAACAGCG
GAPDH-R	ACCACCCTGTTGCTGTAGCCAA

Supplementary Figures:

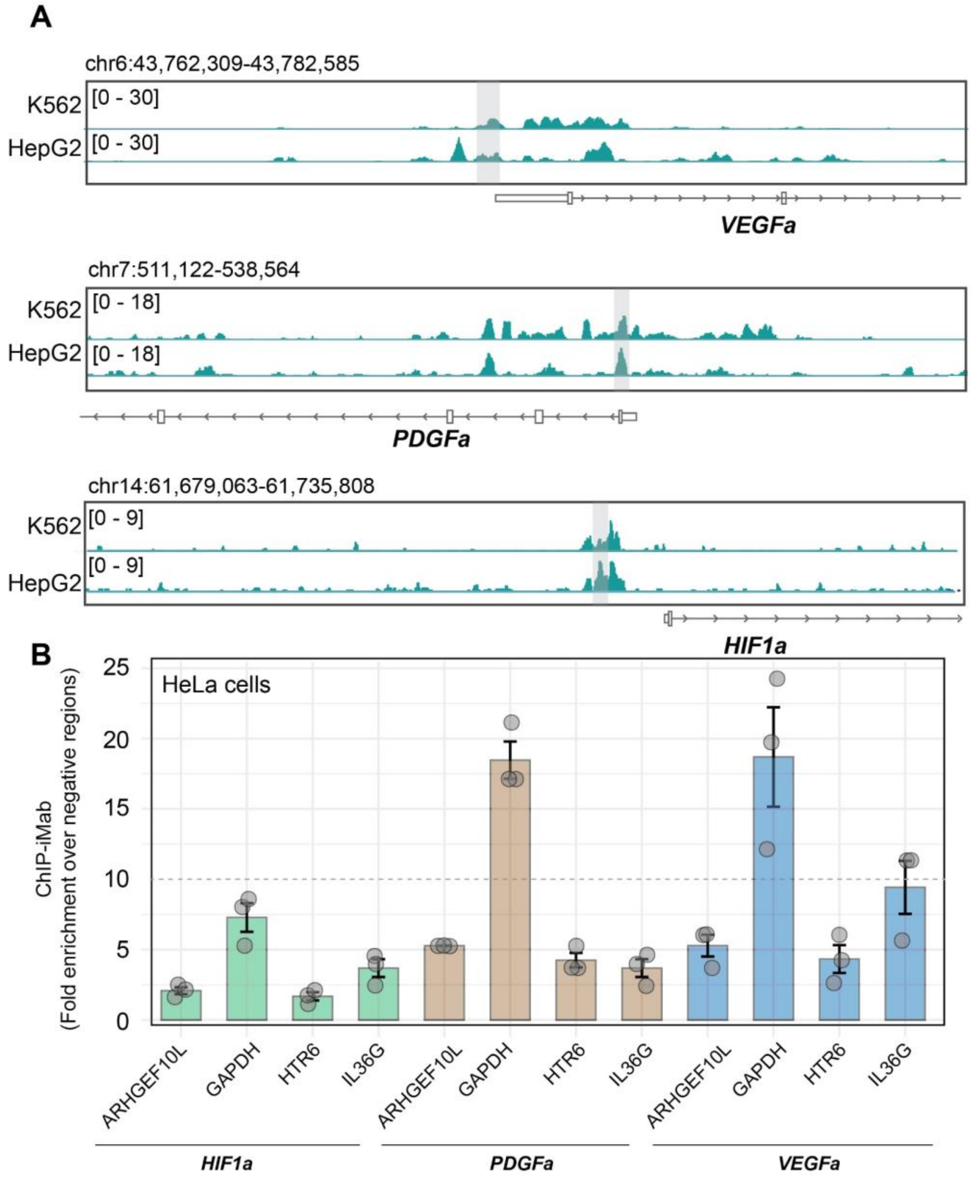

Supplementary Fig. 1. PCBP1 occupancy at C-rich genomic sequences at *VEGFa*, *PDGFa*, and *HIF1α* promoters and their potential to form i-motif structures. (A) IGV tracks visualizing ChIP-seq data of PCBP1-bound regions at *VEGFa*, *PDGFa*, and *HIF1a* promoters in K562 and HepG2 cells. Each track shows the signal intensity range, with a scale noted in brackets for each genomic region, indicating the level of PCBP1 occupancy across those genomic regions for different cell types. The highlighted light grey regions overlap with i-motif-forming regions. **(B)** Quantification of iMab-ChIP efficiency at *VEGFa*, *PDGFa*, and *HIF1a* promoters versus several non-i-motif negative control sites in HeLa cells (*ARHGEF10L, GAPDH, HTR6, IL36G*). The y-axis shows fold enrichment over negative regions. ChIP-qPCR bar plots are represented as means ± SD of triplicates.

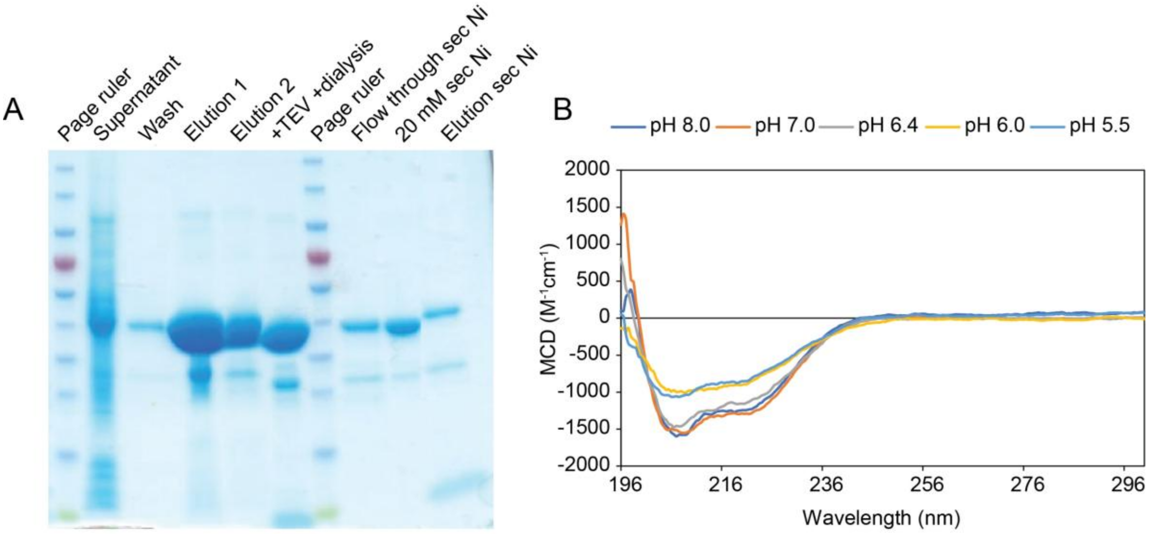

Supplementary Fig. 2. Purification of recombinant PCBP1 and its secondary structures at different pH. (**A**) Recombinant PCBP1 purified from *Escherichia coli* BL21(DE3) cells induced at 18 °C. Supernatant showing soluble fraction after cell lysis, sonication and centrifugation. The supernatant was filtered through a 0.45 µm membrane and loaded onto Thermo Scientific Ni-NTA gravity columns. After washing, PCBP1 was eluted with 300 mM NaCl and 300 mM imidazole, then dialyzed and subjected to TEV (Tobacco Etch Virus) protease cleavage to cleave off the His-tag. The final elution was obtained in a buffer containing 20 mM Tris-HCl (pH 8.0), 150 mM NaCl, 20% glycerol, and 1 mM DTT (Dithiothreitol). (**B**) CD spectra of full-length PCBP1 at 25 ºC in 20 mM Tris, 150 mM NaCl in different pH.

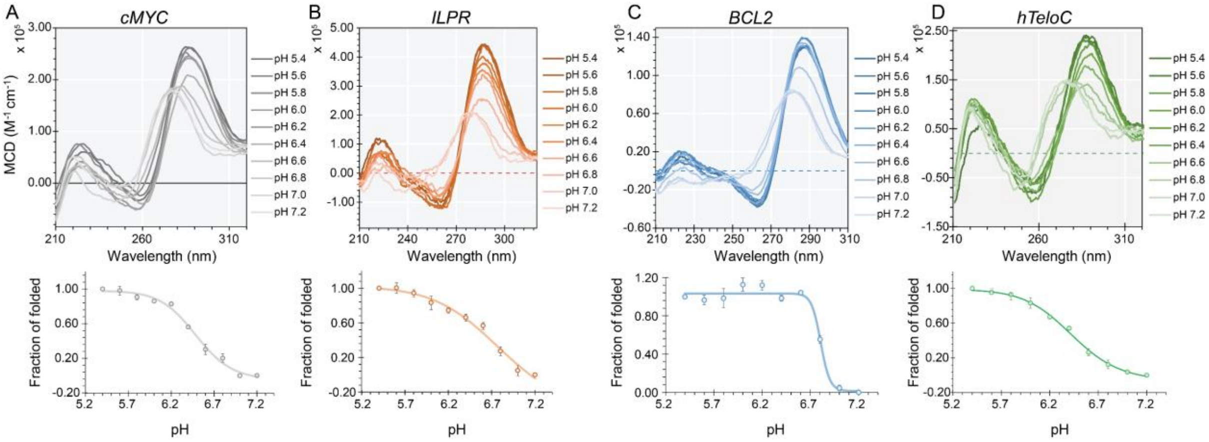

Supplementary Fig. 3. CD melting profiles of **(A)** *cMYC*, **(B)** *ILPR*, **(C)** *BCL2*, and **(D)** hTeloC i-motifs within a broad pH range (5.4-7.2). The positive maxima around 287 nm indicate i-motif structure (*Top*) in the CD spectra of the i-motifs. The sigmoidal curves fitted to the data-points corresponding to the positive maxima of each i-motif at different pH (*Bottom*) to calculate the pH_T or transition pH. Error bars expressed as means ± SD from three biological replicates.

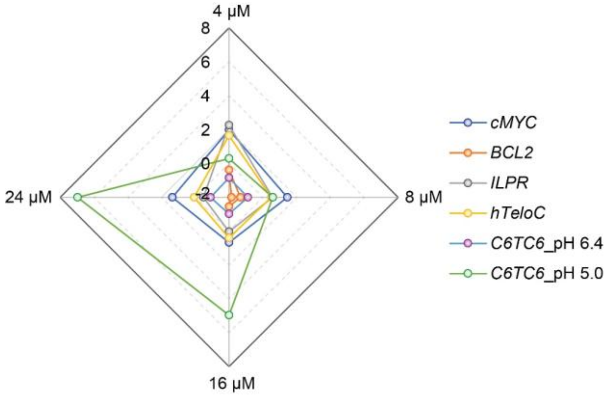

Supplementary Fig. 4. Concentration-dependent CD melting assay. Radar plot showing the changes in melting temperatures (ΔT_m) (in ^{o}C) for *cMYC*, *BCL2*, *ILPR*, and hTeloC, *C6TC6* i-motifs in 10 mM MES buffer (pH 6.4) and 100 mM NaCl in different oligonucleotide concentrations (4, 8, 16, and 24 μM) relative to 2 μM. Radar y-axis denotes ΔT_m values relative to 2 μM. C6TC6 at pH 5.0 was used as control for intermolecular i-motif.

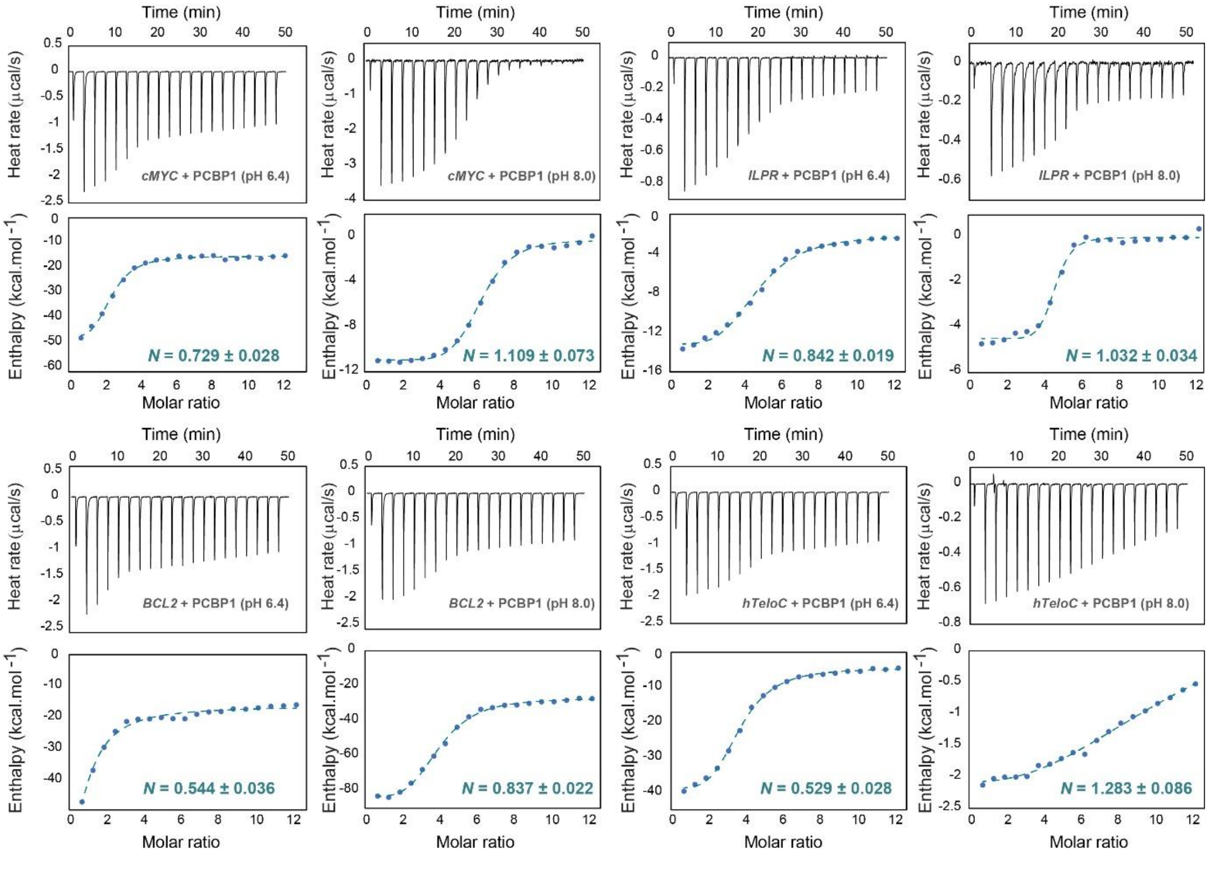

Supplementary Fig. 5. ITC showing intermolecular interactions between PCBP1 and iMfs under pH 6.4 and pH 8.0 at 25 ^{o}C. *Top panels:* enthalpic heat released versus time during titrations. *Bottom panels:* thermogram of the integrated peak intensities plotted against the molar ratio of the complex. Best-fit curves using single-site binding models and number of binding sites are provided as *N* for each binding reaction.

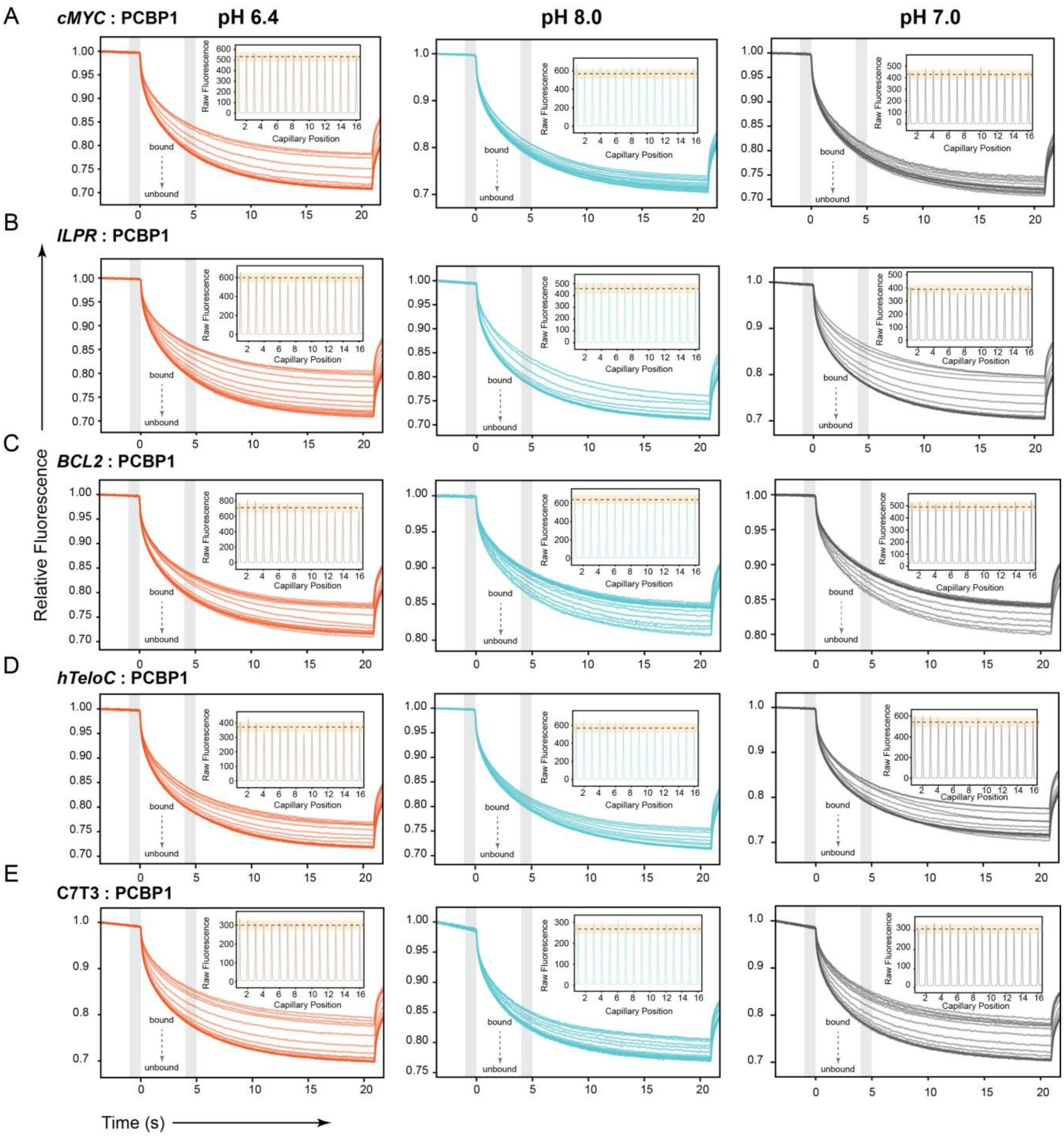

Supplementary Fig. 6. MST traces and raw fluorescence profiles showing the binding interactions between PCBP1 and iMfs (A) *cMYC,* **(B)** *ILPR*, **(C)** *BCL2*, **(D)** hTeloC and **(E)** C7T3 at 25 ºC under three different pH conditions: in 20 mM MES, 100 mM NaCl at pH 6.4 (orange traces), in 20 mM Tris, 100 mM NaCl at pH 8.0 (cyan traces), and in 20 mM sodium phosphate, 100 mM NaCl at pH 7.0 (gray traces). MST traces showing relative fluorescence of the PCBP1-bound and unbound iMfs vs MST experimental time. Insets in each graph display the raw fluorescence as a function of capillary position.

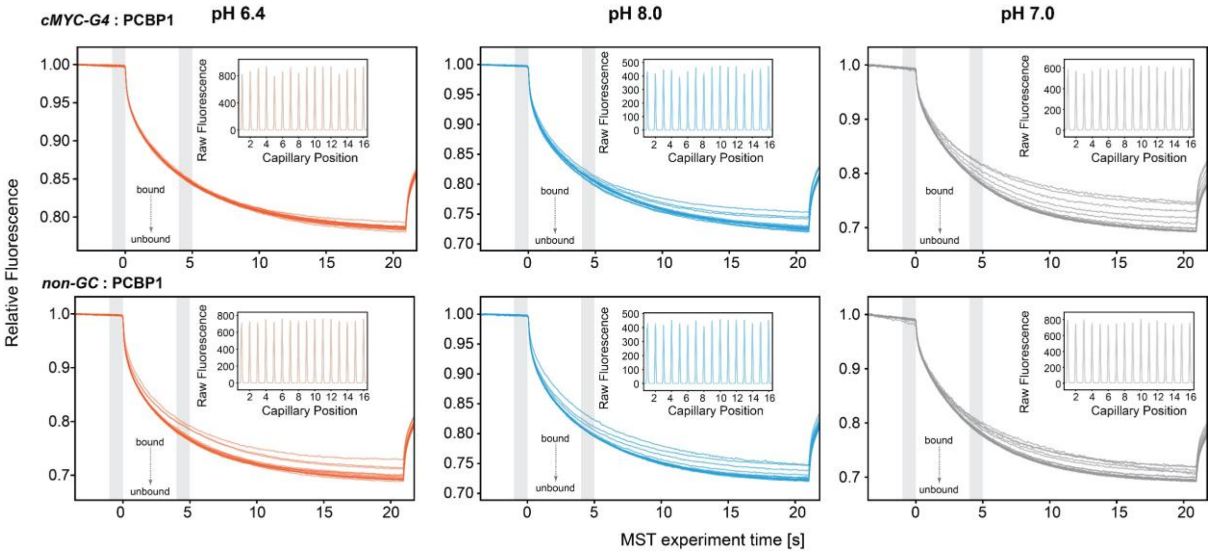

Supplementary Fig. 7. MST traces and raw fluorescence profiles showing the binding interactions between PCBP1 and control sequences (*cMYC-G4* and non-GC) at 25 ºC under three different pH conditions: in 20 mM MES, 100 mM NaCl at pH 6.4 (orange traces), in 20 mM Tris, 100 mM NaCl at pH 8.0 (cyan traces), and in 20 mM sodium phosphate, 100 mM NaCl at pH 7.0 (gray traces). MST traces showing relative fluorescence of the PCBP1-bound and unbound control sequences vs MST experimental time. Insets in each graph display the raw fluorescence as a function of capillary position.

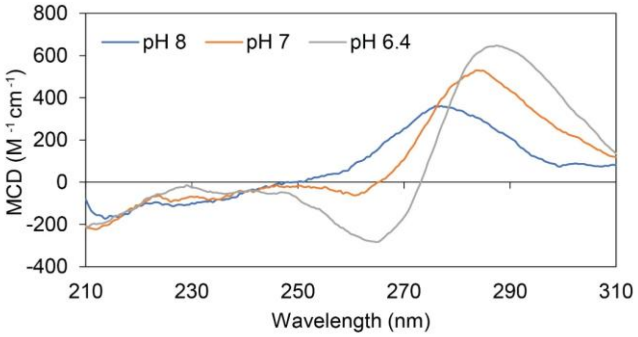

Supplementary Fig. 8. CD spectra of C7T3 in 10 mM MES (pH 6.4) or 10 mM sodium phosphate (pH 7.0), or 10 mM Tris (pH 8.0) buffer supplemented with 100 mM NaCl.

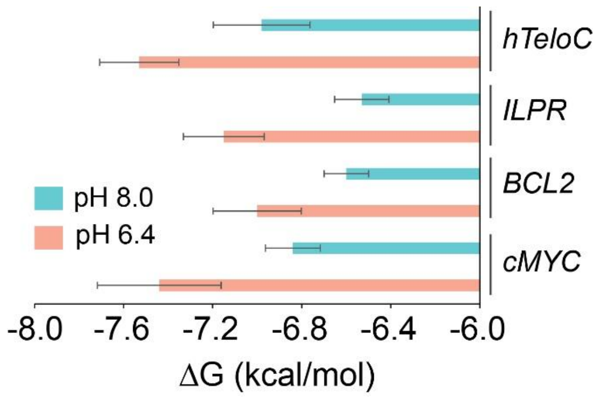

Supplementary Fig. 9: ΔG (difference between binding free energy) between pH 8.0 and pH 6.0 in the binding reactions between PCBP1 and iMfs.

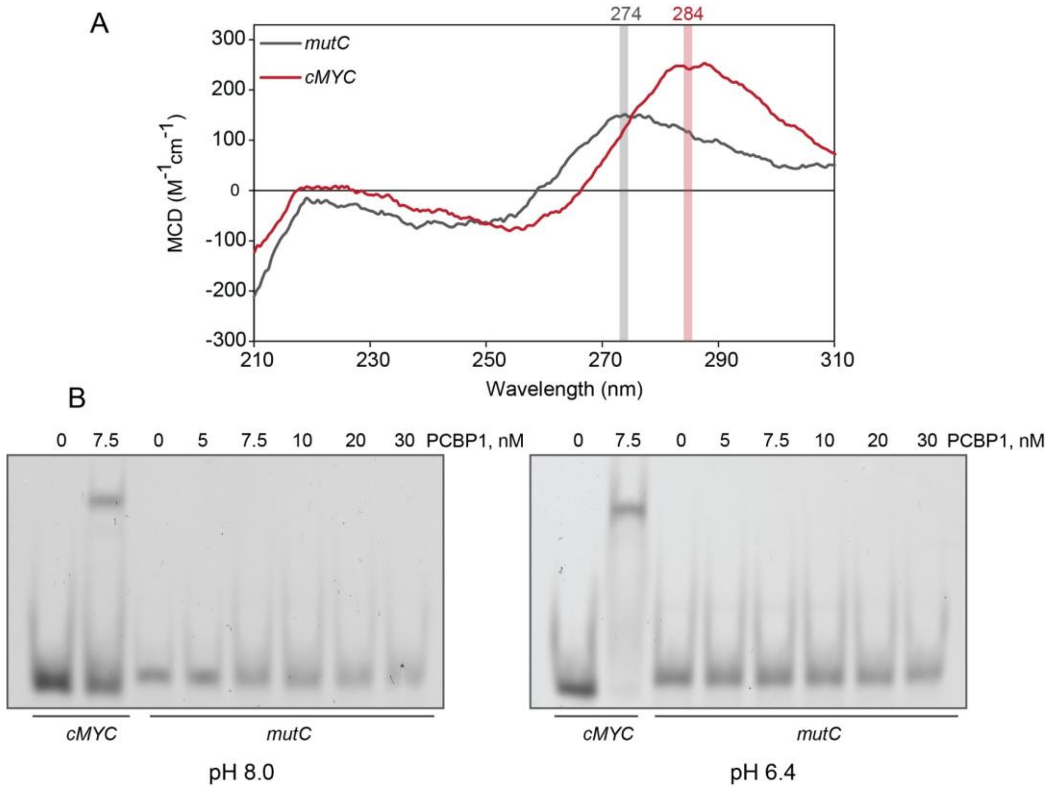

Supplementary Fig. 10: I-motif-forming potential and interaction with PCBP1 by *mutC* sequence. (A) CD spectra of *cMYC* and *mutC* in 10 mM MES buffer (pH 6.4) and 100 mM NaCl at 25 ^{o}C. Red and grey bars represent their respective positive maxima. **(B)** EMSA gels at pH 8.0 and pH 6.4 showing interaction between 5 nM of *mutC* and PCBP1 at increasing concentrations alongside positive control of *cMYC* (5 nM) and *cMYC*-bound to 7.5 nM PCBP1 at pH 8.0 and pH 6.4, respectively.

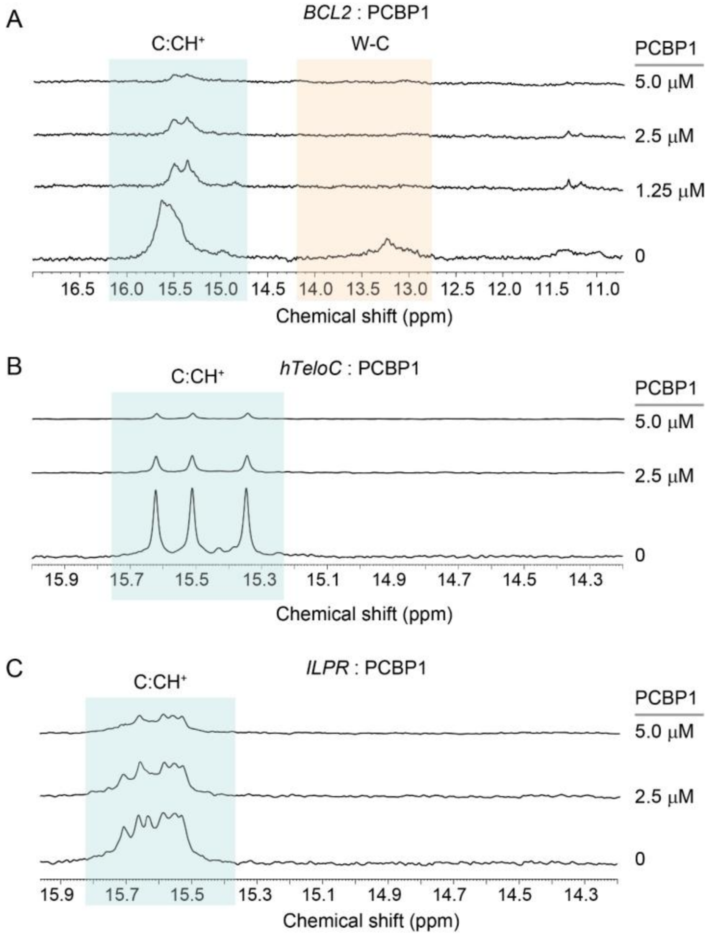

Supplementary Fig. 11. **Changes in the imino proton resonances (15-16 ppm) in the ^{1}H 1D NMR spectra** of **(A)** *BCL2*, **(B)** hTeloC, and **(C)** *ILPR* i-motifs upon titration of various concentrations of PCBP1 at 25 ºC in 20 mM MES, 100 mM NaCl

at pH 6.4. The C:CH$^+$ pairs for i-motif structures (15-16 ppm) and W-C (Watson-Crick) base pairs of hairpin structures (13-14 ppm) highlighted in the ^{1}H 1D NMR spectra.

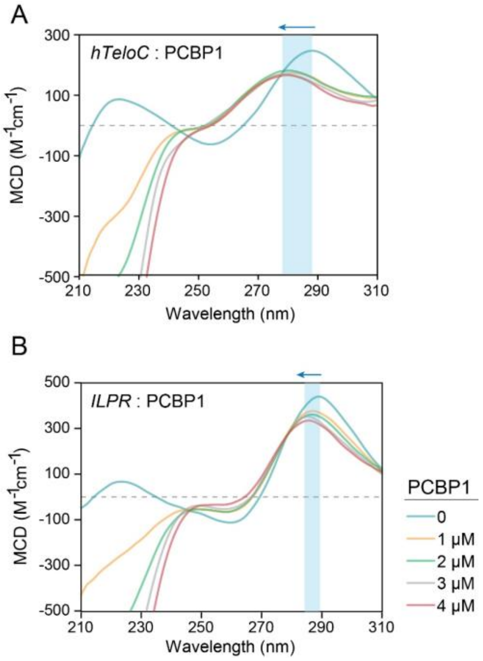

Supplementary Fig. 12. CD spectra of **(A)** hTeloC and **(B)** *ILPR* i-motifs upon PCBP1 titrations at 25 ºC in 20 mM MES, 100 mM NaCl at pH 6.4. The highlighted regions showing blue-shift (with arrow) and hypochromic effects.

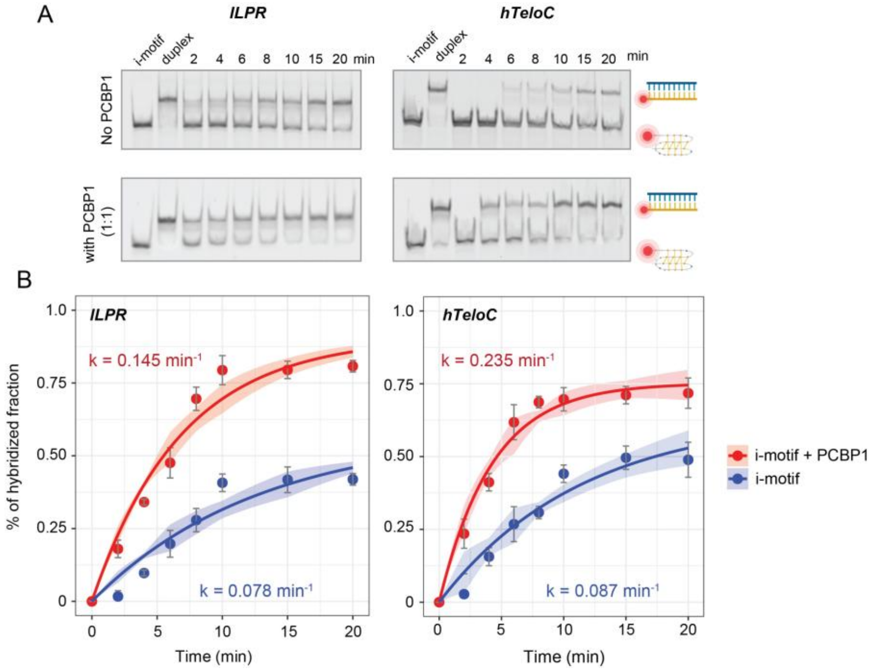

Supplementary Fig. 13. I-motif-destabilization trapping assay in *ILPR* and hTeloC. **(A)** Native PAGE visualizing hybridization reaction aliquots at different time intervals up to 14 min and 20 min respectively for *ILPR* and h*TeloC*, alongside negative control (i-motifs without complementary strands) and positive controls (hybridized duplex). Reactions performed at 25 ºC in 20 mM MES (pH 6.4), 50 mM NaCl, 2 mM $MgCl_2$, and 0.25 mg/mL BSA. **(B)** Densitometric analysis of i-motif-trapping. Percentage intensities of hybridized fractions calculated from three biological replicates at different time points, fitted to mono-exponential function.

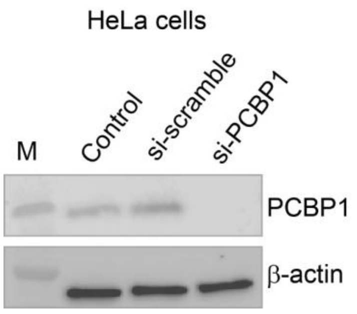

Supplementary Fig. 14. Western blot confirmation of siRNA-mediated PCBP1 knockdown (PCBP1-KD) in HeLa cells. β-actin used as loading reference. M indicates molecular size marker.

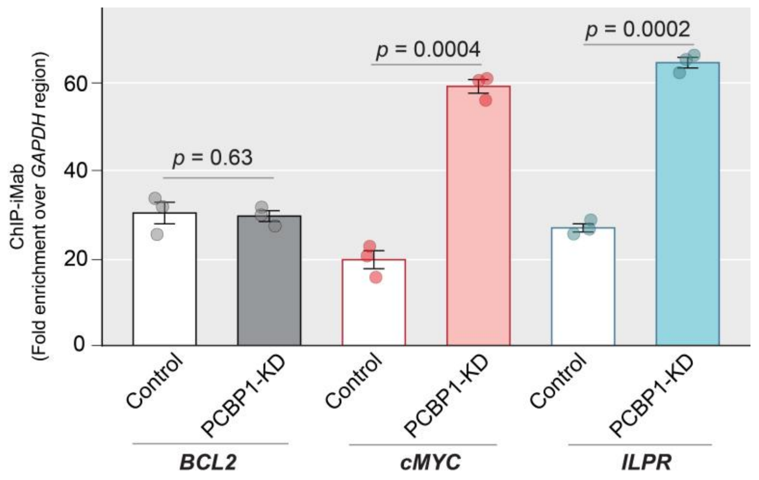

Supplementary Fig. 15. Quantification of iMab-ChIP efficiency at i-motif-harboring oncogene promoters (*cMYC, BCL2, ILPR*) versus non-i-motif control site (*GAPDH*) at pH 7.4, using percentage of input method; error bars from three biological replicates. Statistical differences between control and PCBP-KD determined by one-way ANOVA followed by Tukey–Kramer test (* indicating p-value < 0.05, ** indicating p-value < 0.01, *** indicating p-value < 0.001 and ns indicating non-significant results).

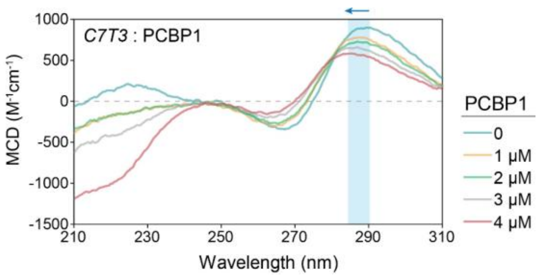

Supplementary Fig. 16. CD spectra of C7T3 i-motif upon different concentrations of PCBP1 titrations at 25 ºC in 20 mM MES, 100 mM NaCl at pH 6.4, with highlighted regions showing blue-shift (with arrows) and hypochromic effects.

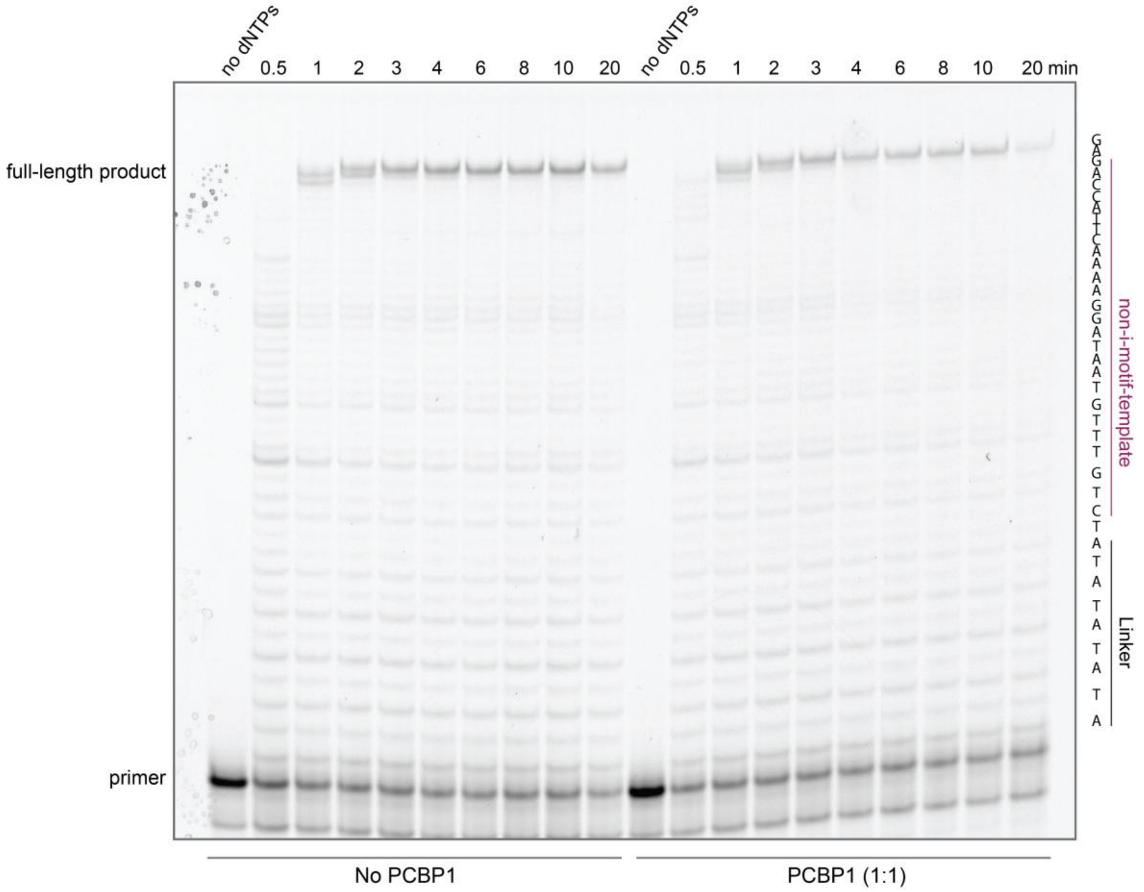

Supplementary Fig. 17. Primer extension assay with non-i-motif template. In presence of 40 nM PCBP1 at different time-points (0.5 – 20 min) alongside 0 min reaction with no PCBP1 and no reaction controls. Reactions performed at 25 ºC in 20 mM MES (pH 6.0), 6 mM $MgCl_2$, 0.2 mg/ml BSA, 0.05 µU/L Klenow fragment, and 0.2 mM dNTPs.

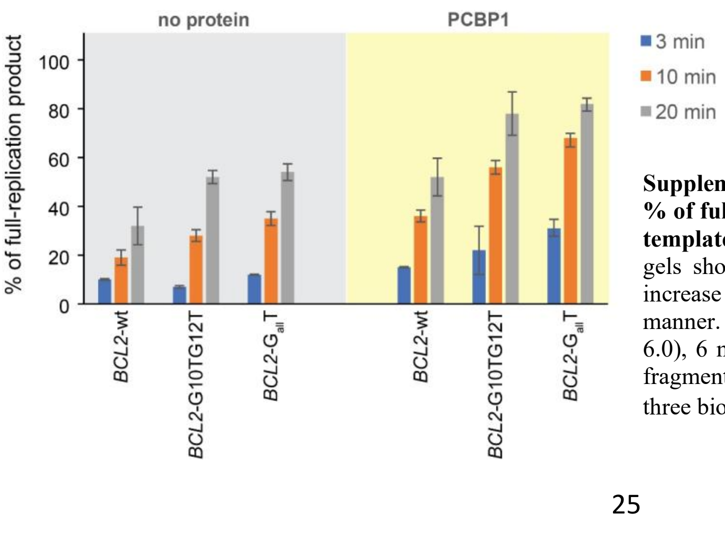

Supplementary Fig. 18. Densitometric analyses of the % of full-replication products in *BCL2*-wt and mutant templates. The densitometric analysis were performed on gels shown in Fig. 4F. In presence of 40 nM PCBP1, increase in full-replication products in time-dependent manner. Reactions performed at 25 ºC in 20 mM MES (pH 6.0), 6 mM $MgCl_2$, 0.2 mg/ml BSA, 0.05 µU/L Klenow fragment, and 0.2 mM dNTPs. Error bars calculated from three biological replicates.

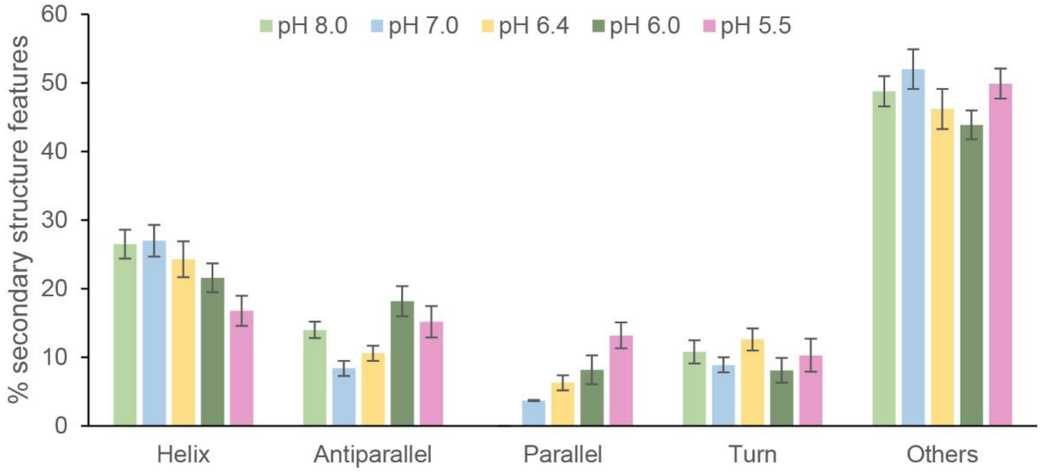

Spectral deviation	pH 8.0	pH 7.0	pH 6.4	pH 6.0	pH 5.5
RMSD	0.1283	0.1921	0.1512	0.1795	0.1898
NRMSD	0.0352	0.0292	0.0195	0.0278	0.0276

Supplementary Fig. 19. Quantitative analysis of PCBP1 secondary structural features (α-helix, β-sheet, turn and others) at different pH values (pH 8.0, 7.0, 6.4, 6.0 and 5.5), calculated from CD spectra using the BeStSel deconvolution algorithm

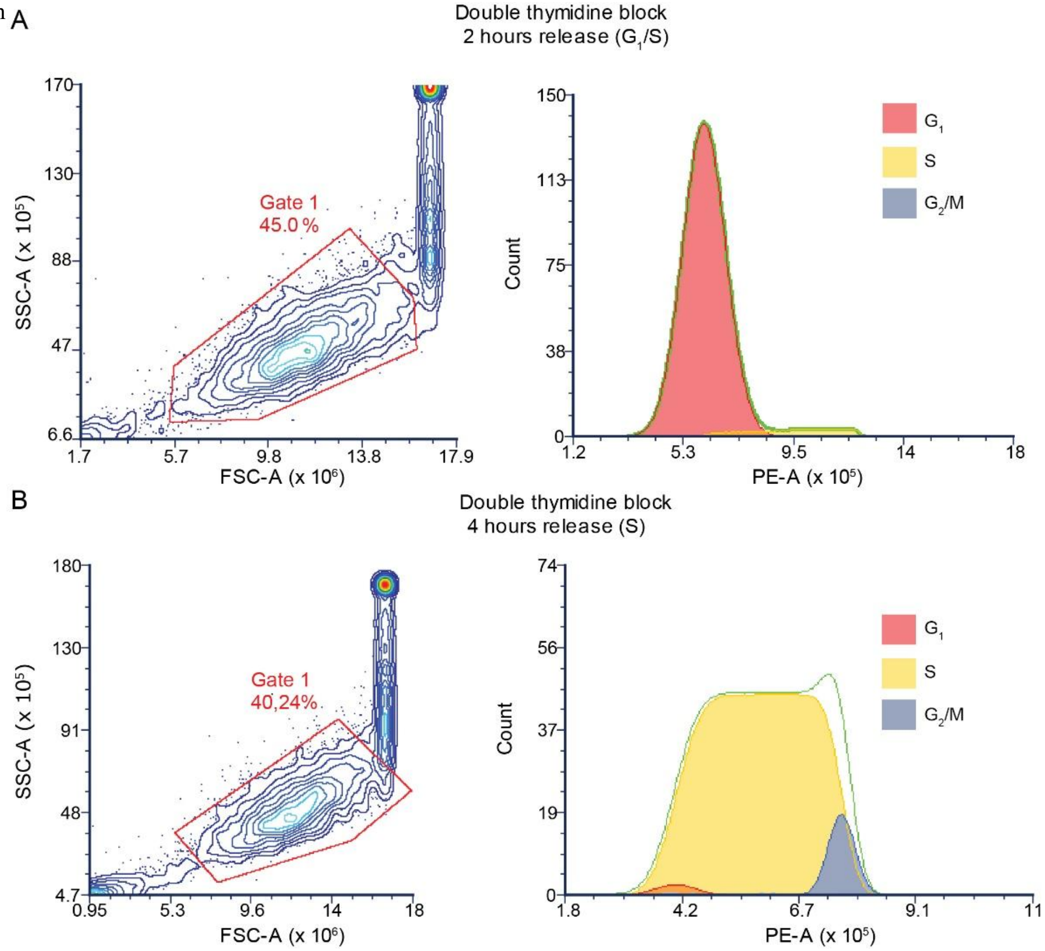

Supplementary Fig 20. Cell-cycle analysis following double thymidine block and release. HeLa cells were synchronized by double thymidine block and collected at the indicated times after release. Representative FSC-A (Forward scatter-area) vs SSC-A (Side scatter area) plots (left) show the gating strategy used to select the main population. DNA content histograms (right) display the distribution of cells in G_1 (red), S (yellow), and G_2/M (blue) phases at 2 h **(A)** and 4 h **(B)** post-release.

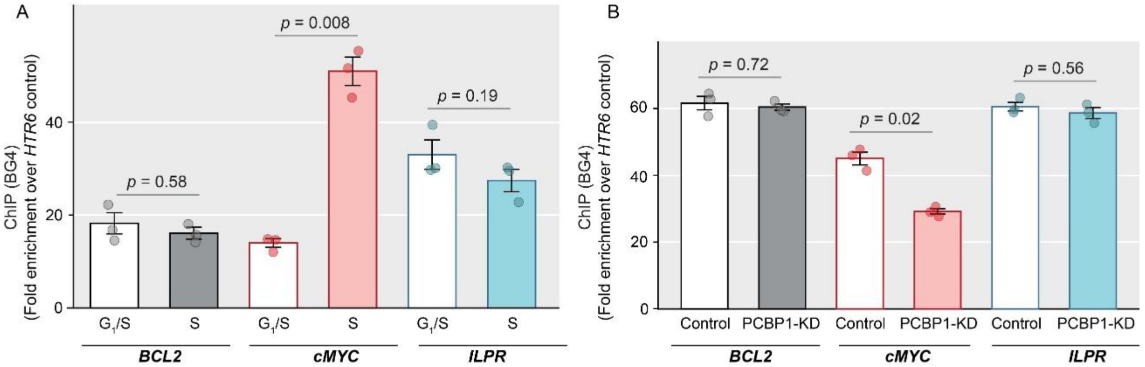

Supplementary Fig. 21. Quantification of BG4-ChIP efficiency at i-motif-harboring oncogene promoters (*cMYC, BCL2, ILPR*) versus non-G4 control site (*HTR6*), using percentage of input method; error bars from three biological replicates. Statistical differences between **(A)** G_1/S and S phase cells and **(B)** Control and PCBP1-KD cells were determined by one-way ANOVA followed by Tukey–Kramer test.

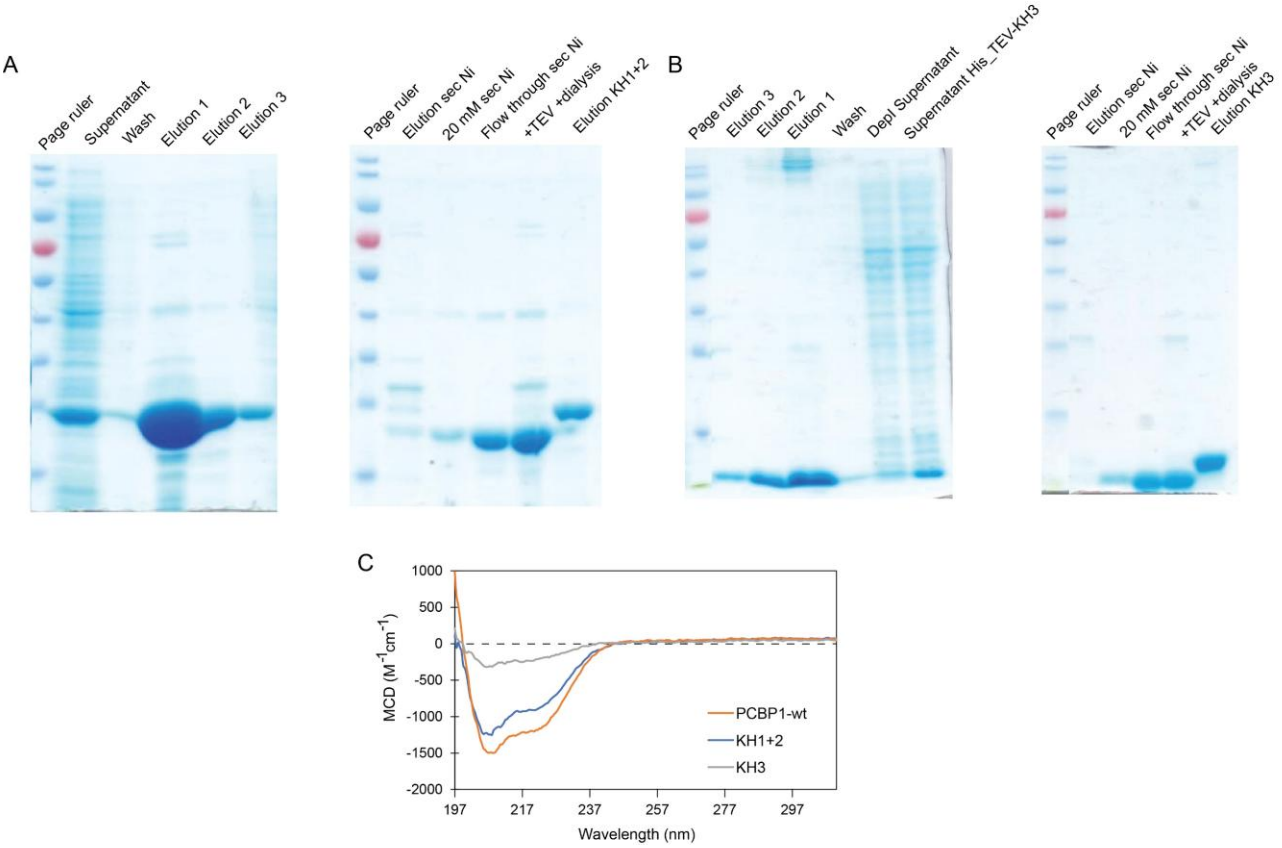

Supplementary Fig. 22. Purification of (A) KH1+2 and (B) KH3 domains of PCBP1. Recombinant KH mutants purified from *Escherichia coli* BL21(DE3) cells induced at 20 °C. Supernatant showing soluble fraction after cell lysis, sonication and centrifugation. The supernatant was filtered through a 0.45 µm membrane and loaded onto Thermo Scientific Ni-NTA gravity columns. After washing, KH mutants were eluted with 300 mM NaCl and 300 mM imidazole, then dialyzed and subjected to TEV protease cleavage to cleave off the His-tag. The final elution was obtained in a buffer containing 20 mM Tris-HCl (pH 8.0), 150 mM NaCl, 20% glycerol, and 1 mM DTT. (**C**) CD spectral profiles of 4 µM of purified PCBP1 and the KH-mutants (KH1+2 and KH3) in 20 mM Tris-HCl (pH 8.0), 150 mM NaCl, 20% glycerol, and 1 mM DTT at 25 ^{o}C.

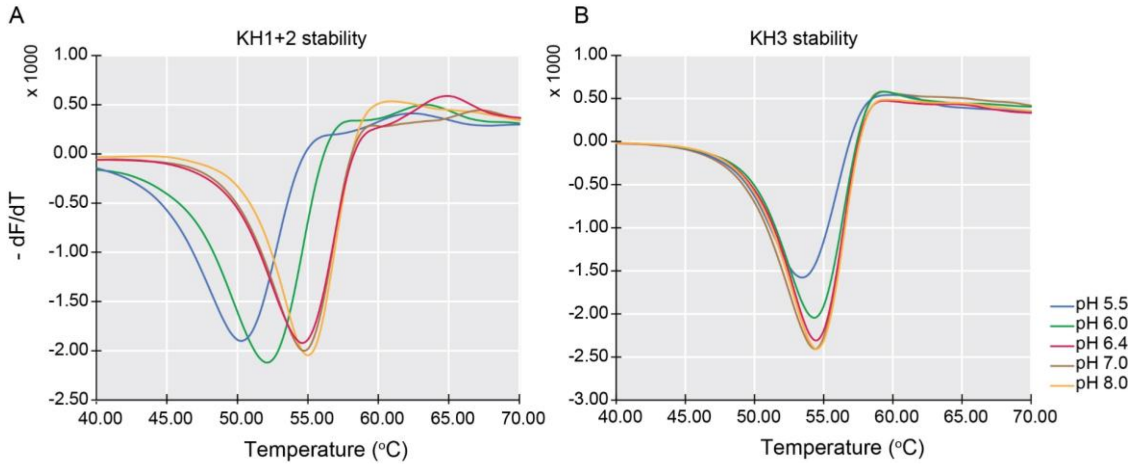

Supplementary Fig. 23. Thermal stability of KH mutants. Thermal-shift assay of **(A)** KH1+2 and **(B)** KH3, with $-\frac{dF}{dT}$ representing the negative derivative of SYPRO-Orange fluorescence signal with respect to temperature; T_m defined by negative maxima at different pH values. Assays performed in 20 mM MES buffer at pH 5.5, 6.0, 6.4 and in 20 mM Tris-Cl buffer at pH 7.0 and 8.0, supplemented with 100 mM NaCl.

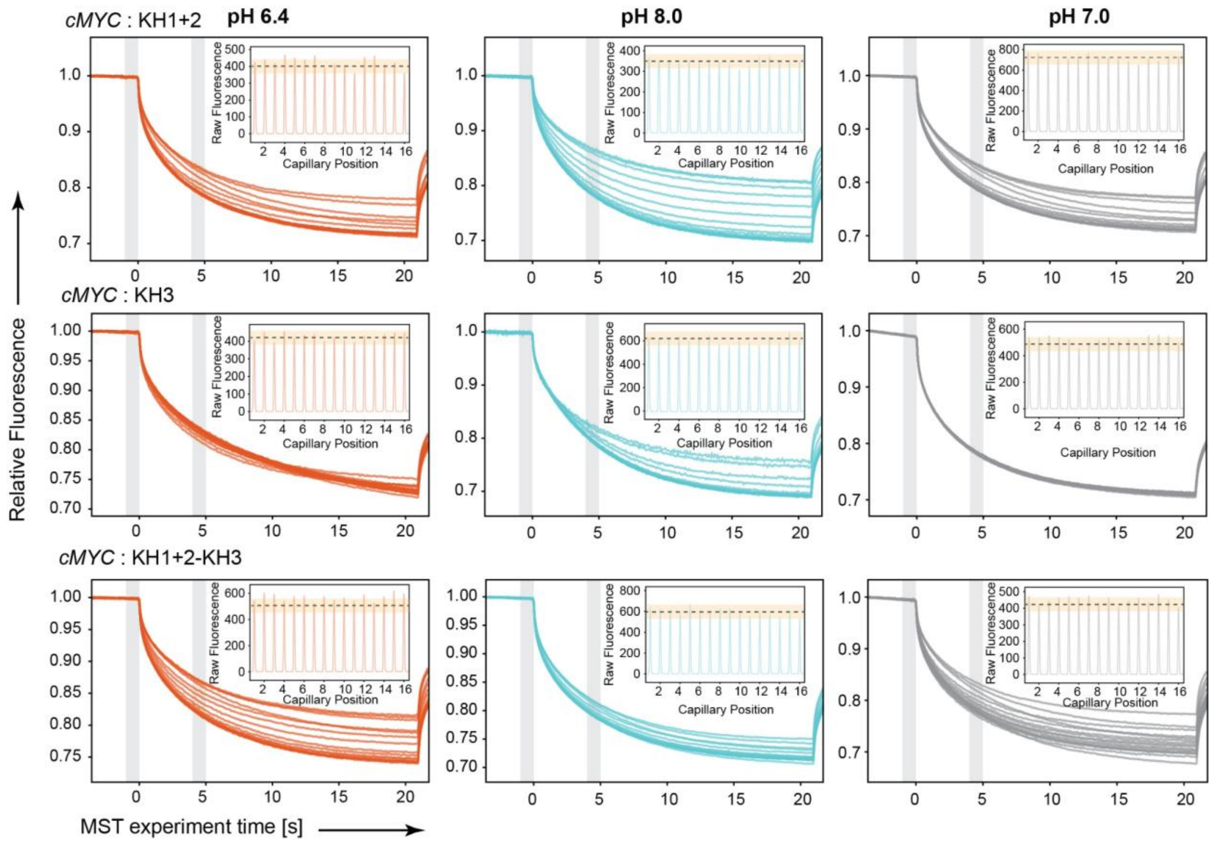

Supplementary Fig. 24. MST traces and raw fluorescence profiles showing the binding interactions between KH-mutants and *cMYC*-iMfs at 25 ºC under three different pH conditions: in 20 mM MES, 100 mM NaCl at pH 6.4 (orange traces), in 20 mM Tris, 100 mM NaCl at pH 8.0 (cyan traces), and in 20 mM sodium phosphate, 100 mM NaCl at pH 7.0 (gray traces). MST traces showing relative fluorescence of the KH-mutants-bound and unbound cMYC-iMfs vs MST experimental time. Insets in each graph display the raw fluorescence as a function of capillary position.

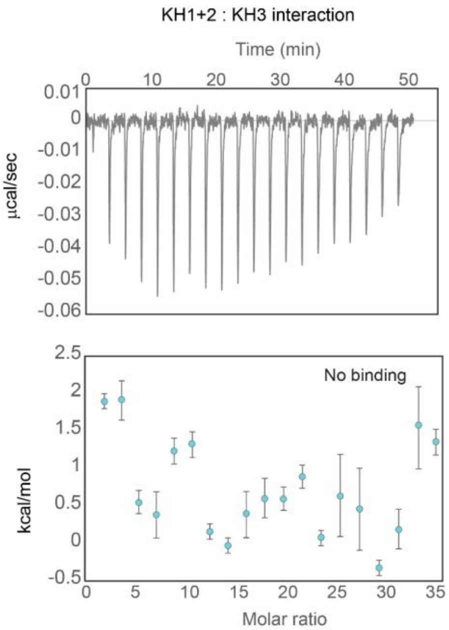

Supplementary Fig. 25. ITC profile showing intermolecular interactions between KH1+2 and KH3. *Top panel*: enthalpic heat released versus time at 25ºC in 20 mM MES (pH 6.4), 100 mM NaCl during titrations. *Bottom panel*: thermogram of the integrated peak intensities plotted against molar ratio.

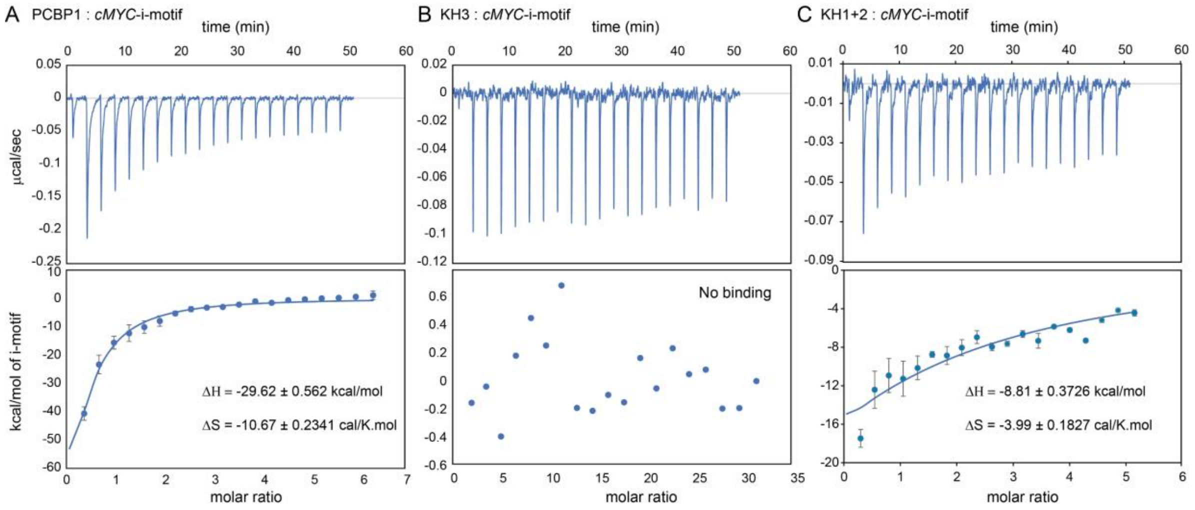

Supplementary Fig. 26. ITC profile showing intermolecular interactions between PCBP1 and KH mutant proteins and *cMYC*-i-motif at pH 6.4. (A) PCBP1-*cMYC*-i-motif interaction**. (B)** KH3 mutant-*cMYC*-i-motif interaction**. (C)** KH1+2 mutant-*cMYC*-i-motif interaction. *Top*: enthalpic heat released versus time at 25ºC in 20 mM MES (pH 6.4), 100 mM NaCl during titrations. *Bottom*: thermogram of the integrated peak intensities plotted against molar ratio. ΔH and ΔS calculated based on one-site binding model using integrated software and indicate enthalpic and entropic contributions of binding. Error bars indicate three biological replicates.

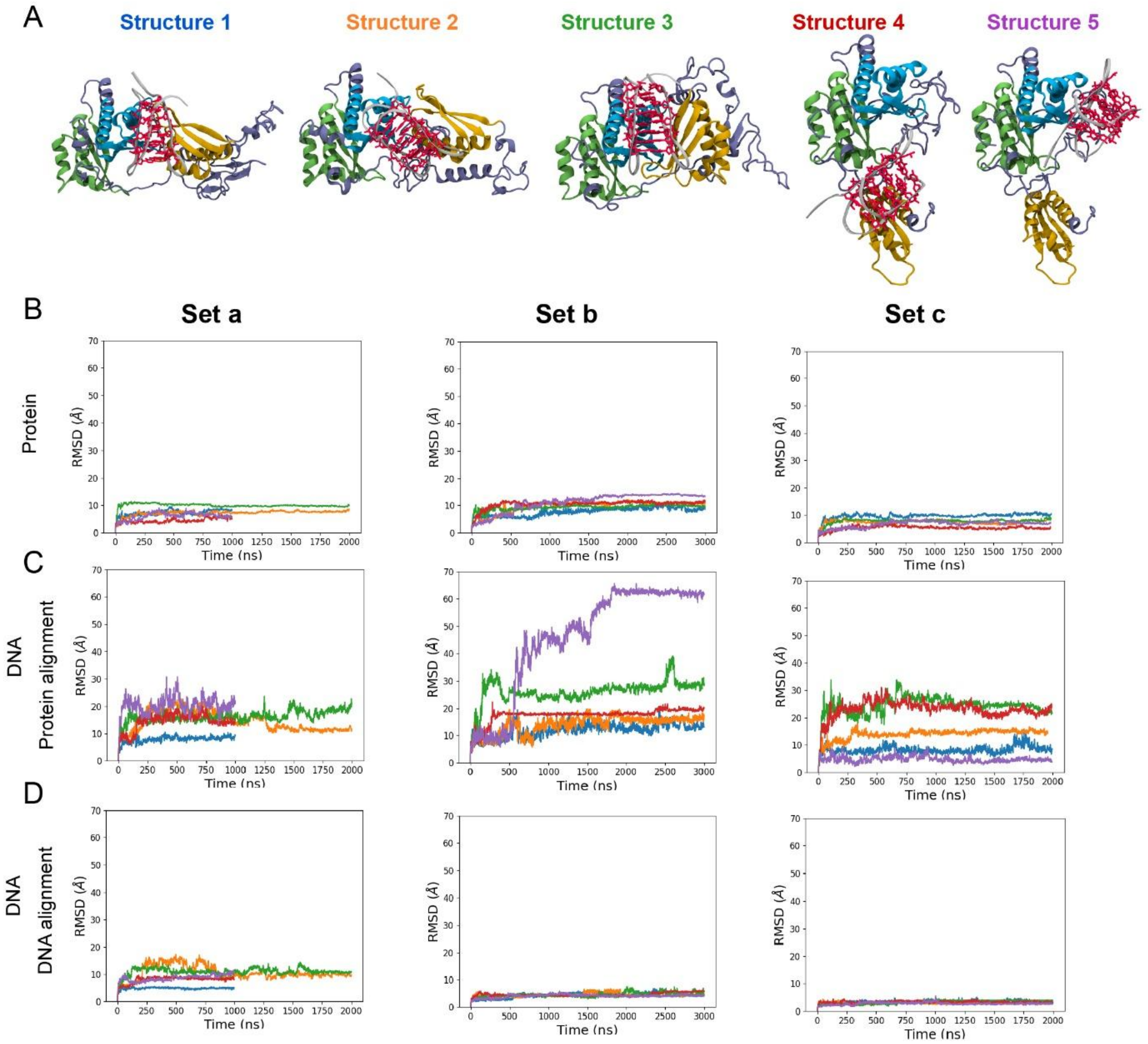

Supplementary Fig. 27. ***In silico*** **modelling of PCBP1-*ILPR*-i-motif complexes (A)** Five starting structures for MD simulations with *ILPR*-i-motif backbone (silver), cytosines (red), KH1 domain (cyan), KH2 domain (light green), KH3 domain (yellow), interconnecting domains (violet). RMSD evolution along the MD simulations for the five structures (1: blue; 2: orange; 3: green; 4: red; 5: purple) in set a, b and c for **(B)** the whole protein**, (C)** DNA after alignment of the trajectories on the protein position and **(D)** DNA after alignment on DNA position.

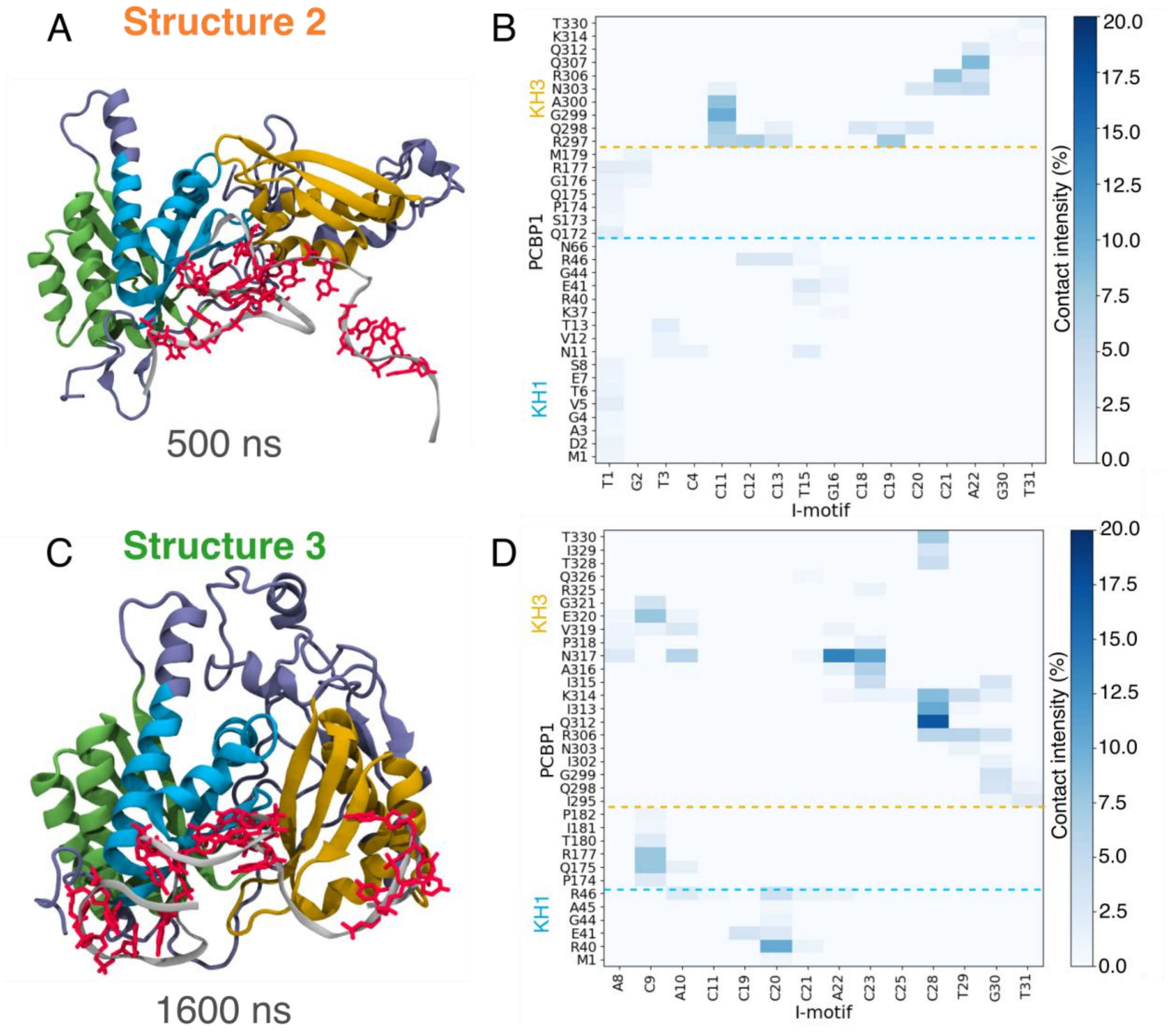

Supplementary Fig. 28. Unfolding event in Molecular Dynamics simulations in set (a). (A) and (C) Structures 2 and 3 from set (a) from molecular dynamics simulation with a partial unfold of the *ILPR*-i-motif. **(B)** and **(D)** Contact heatmap between PCBP1 and *ILPR*-i-motif for the 3 µs molecular dynamics simulation; a contact is counted with one heavy atom of an amino-acid is at less than 4 Å for structure 2 (B) or 3 (D).

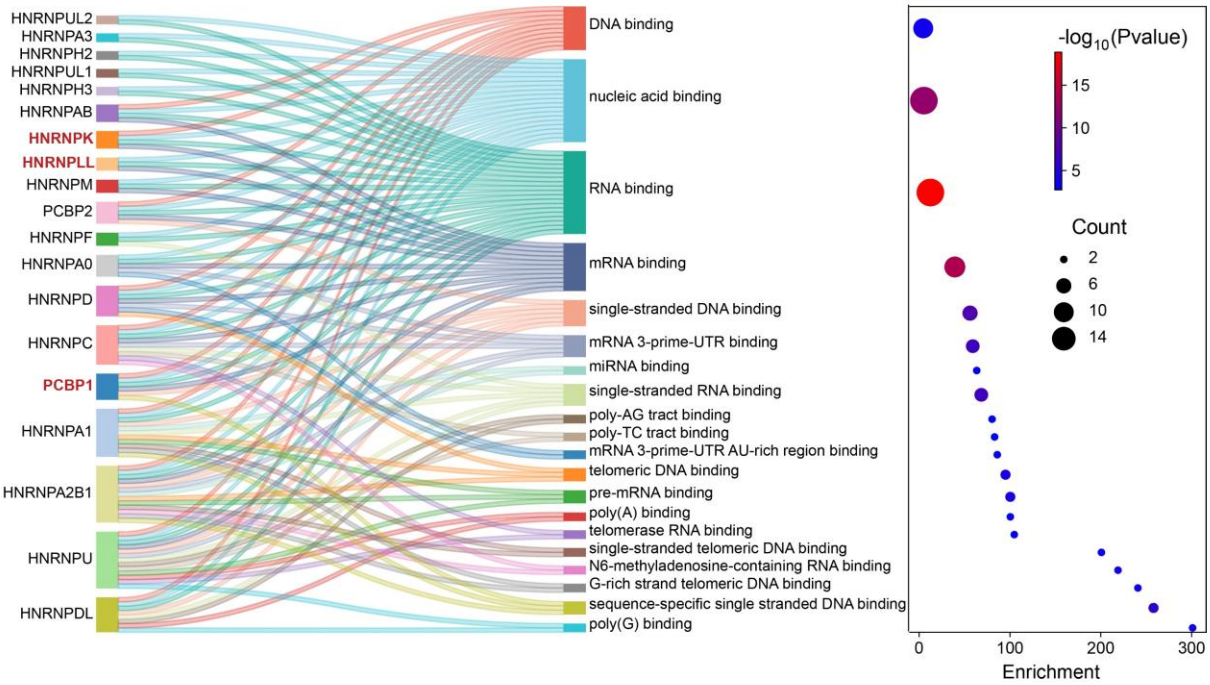

Supplementary Fig. 29: GO (Gene Ontology) enrichment analyses of biological processes (BP) regulated by HNRNP family proteins that are identified to potentially interact with i-motif in the proteomic study by Ban et al. The Sankey plot on the left side represents overlapping functions of different hnRNP proteins in nucleic acid binding. On the right shows the gene enrichment bubble plot. Enrichment significance is represented by -log10(Pvalue). The higher the enrichment score value is (more red), the more significant the pathway is.

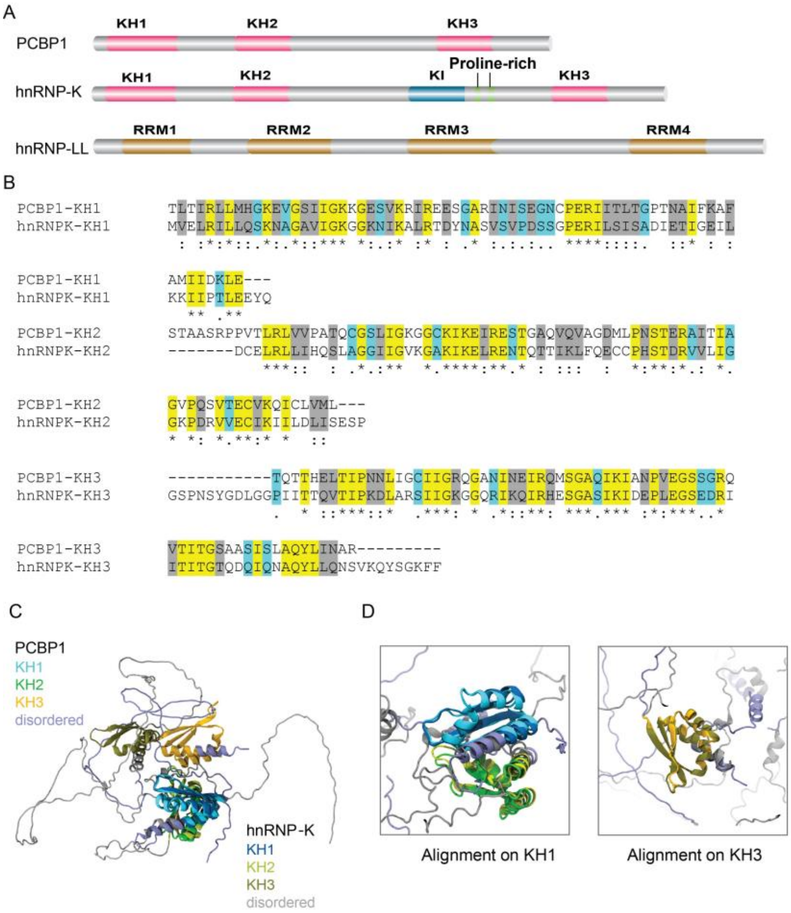

Supplementary Fig. 30: Different type of nucleic acid binding domains in PCBP1, hnRNP-LL, and hnRNP-K. **(A)** PCBP1 and hnRNP-K contain three KH domains, while hnRNP-K contains an additional KI and proline-rich domain. hnRNP-LL contains four RRM domains. **(B)** Multiple sequence alignment between the KH domains of hnRNP-K and PCBP1 using clustalW. Amino acids highlighted in yellow and marked with "*" are similar between both proteins. Amino acids highlighted in grey and marked with ":" are not identical but have similar properties between both proteins. Amino acids highlighted in blue and marked with . are non-identical and share less properties between both proteins. Amino acids that are not marked or blank are different between both proteins. **(C)** Overlay of alphafold-predicted structural models of PCBP1 and hnRNP-K with their respective KH domains and disordered regions marked. **(D)** Alignment on KH1 shows overlay of KH1 and KH2 with highly conserved folds; separate alignment on KH3 likewise shows a well-conserved KH3 domain. The disordered linkers between KH domains differ markedly between the two proteins.

Supplementary References:

1. Sengupta, P., Jamroskovic, J. & Sabouri, N. A beginner's handbook to identify and characterize i-motif DNA. in *Methods in Enzymology* vol. 695 45–70 (Elsevier, 2024).
2. Micsonai, A. *et al.* BeStSel: a web server for accurate protein secondary structure prediction and fold recognition from the circular dichroism spectra. *Nucleic Acids Research* **46**, W315–W322 (2018).
3. Bag, S. *et al.* Exploring i-Motif DNA binding with benzothiazolino Coumarins: Synthesis, Screening, and spectroscopic insights. *Bioorganic Chemistry* **156**, 108227 (2025).
4. Abdelhamid, M. A. S. & Waller, Z. A. E. Tricky Topology: Persistence of Folded Human Telomeric i-Motif DNA at Ambient Temperature and Neutral pH. *Front. Chem.* **8**, 40 (2020).
5. Rajendran, A., Nakano, S. & Sugimoto, N. Molecular crowding of the cosolutes induces an intramolecular i-motif structure of triplet repeat DNA oligomers at neutral pH. *Chem. Commun.* **46**, 1299 (2010).
6. Li, T. & Famulok, M. I-Motif-Programmed Functionalization of DNA Nanocircles. *J. Am. Chem. Soc.* **135**, 1593–1599 (2013).
7. Ross, S. A. & Burrows, C. J. Cytosine-specific chemical probing of DNA using bromide and monoperoxysulfate. *Nucleic Acids Research* **24**, 5062–5063 (1996).
8. Maxam, A. M. & Gilbert, W. [57] Sequencing end-labeled DNA with base-specific chemical cleavages. in *Methods in Enzymology* vol. 65 499–560 (Elsevier, 1980).
9. Jamroskovic, J., Deiana, M. & Sabouri, N. Probing the folding pathways of four-stranded intercalated cytosine-rich motifs at single base-pair resolution. *Biochimie* **199**, 81–91 (2022).
10. Jumper, J. *et al.* Highly accurate protein structure prediction with AlphaFold. *Nature* **596**, 583–589 (2021).
11. Baek, M. *et al.* Accurate prediction of protein structures and interactions using a three-track neural network. *Science* **373**, 871–876 (2021).
12. Baek, M. *et al.* Accurate prediction of protein–nucleic acid complexes using RoseTTAFoldNA. *Nat Methods* **21**, 117–121 (2024).
13. Maier, J. A. *et al.* ff14SB: Improving the Accuracy of Protein Side Chain and Backbone Parameters from ff99SB. *J. Chem. Theory Comput.* **11**, 3696–3713 (2015).
14. Ivani, I. *et al.* Parmbsc1: a refined force field for DNA simulations. *Nat Methods* **13**, 55–58 (2016).
15. Yoo, J. & Aksimentiev, A. New tricks for old dogs: improving the accuracy of biomolecular force fields by pair-specific corrections to non-bonded interactions. *Phys. Chem. Chem. Phys.* **20**, 8432–8449 (2018).
16. Song, D., Luo, R. & Chen, H.-F. The IDP-Specific Force Field *ff14IDPSFF* Improves the Conformer Sampling of Intrinsically Disordered Proteins. *J. Chem. Inf. Model.* **57**, 1166–1178 (2017).
17. Olsson, M. H. M., Søndergaard, C. R., Rostkowski, M. & Jensen, J. H. PROPKA3: Consistent Treatment of Internal and Surface Residues in Empirical p K_a Predictions. *J. Chem. Theory Comput.* **7**, 525–537 (2011).

18. Roe, D. R. & Cheatham, T. E. PTRAJ and CPPTRAJ: Software for Processing and Analysis of Molecular Dynamics Trajectory Data. *J. Chem. Theory Comput.* **9**, 3084–3095 (2013).

19. Guneri, D. *et al.* Structural insights into i-motif DNA structures in sequences from the insulin-linked polymorphic region. *Nat Commun* **15**, 7119 (2024).

20. Humphrey, W., Dalke, A. & Schulten, K. VMD: Visual molecular dynamics. *Journal of Molecular Graphics* **14**, 33–38 (1996).

21. Ban, Y. *et al.* Profiling of i-motif-binding proteins reveals functional roles of nucleolin in regulation of high-order DNA structures. *Nucleic Acids Research* **52**, 13530–13543 (2024).

22. Deiana, M. *et al.* A new G-quadruplex-specific photosensitizer inducing genome instability in cancer cells by triggering oxidative DNA damage and impeding replication fork progression. *Nucleic Acids Research* **51**, 6264–6285 (2023).
