# Supplementary material for: Mechanistic insights into PCBP1-driven unfolding of selected i-motif DNA at G1/S checkpoint
Source: Nat Commun. 2026 Feb 2;17:1149. doi: 10.1038/s41467-026-68822-5 (PMC12865031; doi:10.1038/s41467-026-68822-5)
Supplement: Supplementary file 2 — Description of Additional Supplementary Files [file 41467_2026_68822_MOESM2_ESM.pdf]

Description of Additional Supplementary Files

Supplementary Data 1: Raw ChIP–qPCR C_t values and derived enrichment analysis for iMab ChIP. Raw C_t values obtained from ChIP–qPCR experiments using the iMab antibody at selected genomic loci. Data include input, iMab immunoprecipitation (IP), and mock IgG of target and control samples. Experiments were performed with three independent biological replicates, each analyzed with three technical replicates. Target regions analyzed correspond to promoter or regulatory regions of *cMYC, BCL2, ILPR, VEGFa, PDGFa*, and *HIF1a*, while *ARHGEF10L, HTR6, GAPDH*, and *IL36* served as control regions. Raw C_t values were first averaged across technical replicates, followed by calculation of % input enrichment for each biological replicate. Enrichment values for target regions were then normalized to the corresponding control regions to obtain fold enrichment (target/control). These data correspond to Fig. 1E and Supporting Fig. 1B.

Supplementary Data 2: Raw ChIP–qPCR C_t values and derived enrichment analysis for iMab ChIP following PCBP1 knockdown. Raw C_t values obtained from ChIP–qPCR experiments using the iMab antibody following PCBP1 knockdown (PCBP1-KD). Data include input, iMab-IP, and mock IgG on target and control samples. Experiments were performed with three independent biological replicates, each analyzed with three technical replicates. Target regions analyzed correspond to *cMYC, BCL2*, and *ILPR*, with *HTR6* and *GAPDH* serving as control regions. Raw C_t values were first averaged across technical replicates, followed by calculation of % input enrichment for each biological replicate. Enrichment values for target regions were subsequently normalized to the corresponding control regions to obtain fold enrichment (target/control). These data correspond to Fig. 3F and Supporting Fig. 15.

Supplementary Data 3: Raw ChIP–qPCR C_t values and derived enrichment analysis for PCBP1 ChIP across cell-cycle phases. Raw C_t values obtained from ChIP–qPCR experiments using the PCBP1 antibody in HeLa cells under asynchronous, G_1/S-arrested, and S-phase–arrested conditions. Data include input, PCBP1-IP, and mock IgG on target and control samples. Experiments were performed with three independent biological replicates, each analyzed with three technical replicates. Target regions analyzed correspond to *cMYC, BCL2*, and *ILPR*, with *HTR6* and *GAPDH* serving as control regions. Raw C_t values were first averaged across technical replicates, followed by calculation of % input enrichment for each biological replicate. Enrichment values for target regions were subsequently normalized to the corresponding control regions to obtain fold enrichment (target/control). These data correspond to Fig. 5F and 1G.

Supplementary Data 4: Raw ChIP–qPCR C_t values and derived enrichment analysis for iMab ChIP upon cell cycle synchronisation. Raw C_t values obtained from ChIP–qPCR experiments using the iMab antibody in cell-cycle–synchronized HeLa cells at the G_1/S boundary and S phase. Data include input, iMab-IP, and mock IgG on target and control

samples. Experiments were performed with three independent biological replicates, each analyzed with three technical replicates. Target regions analyzed correspond to *cMYC, BCL2*, and *ILPR*, with *HTR6* and *GAPDH* serving as control regions. Raw C_t values were first averaged across technical replicates, followed by calculation of % input enrichment for each biological replicate. Enrichment values for target regions were subsequently normalized to the corresponding control regions to obtain fold enrichment (target/control). These data correspond to Fig. 5G.

Supplementary Data 5: Raw ChIP–qPCR C_t values and derived enrichment analysis for BG4 ChIP upon cell cycle synchronisation. Raw C_t values obtained from ChIP–qPCR experiments using the BG4 antibody in cell-cycle–synchronized HeLa cells at the G_1/S boundary and S phase. Data include input, BG4-IP, and mock IgG on target and control samples. Experiments were performed with three independent biological replicates, each analyzed with three technical replicates. Target regions analyzed correspond to *cMYC, BCL2*, and *ILPR*, with *HTR6* and *GAPDH* serving as control regions. Raw C_t values were first averaged across technical replicates, followed by calculation of % input enrichment for each biological replicate. Enrichment values for target regions were subsequently normalized to the corresponding control regions to obtain fold enrichment (target/control). These data correspond to Supporting Fig. S21A.

Supplementary Data 6: Raw ChIP–qPCR C_t values and derived enrichment analysis for BG4 ChIP following PCBP1 knockdown. Raw C_t values obtained from ChIP–qPCR experiments using the BG4 antibody following PCBP1 knockdown (PCBP1-KD). Data include input, BG4-IP, and mock IgG on target and control samples. Experiments were performed with three independent biological replicates, each analyzed with three technical replicates. Target regions analyzed correspond to *cMYC, BCL2*, and *ILPR*, with *HTR6* serving as control regions. Raw C_t values were first averaged across technical replicates, followed by calculation of % input enrichment for each biological replicate. Enrichment values for target regions were subsequently normalized to the corresponding control regions to obtain fold enrichment (target/control). These data correspond to Supporting Fig. S21B.

Supplementary Movie 1: Molecular dynamics simulation of PCBP1–*ILPR*-i-motif complex for 2500 ns. The video depicts the structural evolution of structure 1 under set (a) unprotonated condition. *ILPR*-i-motif backbone (silver), unprotonated cytosines (red), KH1 domain (cyan), KH2 domain (light green), KH3 domain (yellow), interconnecting domains (violet). Progressive unfolding events, base-pair disruptions, or stabilization features can be observed over the course of the 2500 ns simulation.

Supplementary Movie 2: Molecular dynamics simulation of PCBP1–*ILPR*-i-motif complex for 2500 ns. The video depicts the structural evolution of structure 3 under set (a) unprotonated condition. *ILPR*-i-motif backbone (silver), unprotonated cytosines (red), KH1 domain (cyan), KH2 domain (light green), KH3 domain (yellow), interconnecting domains (violet). Progressive unfolding events, base-pair disruptions, or stabilization features can be observed over the course of the 2500 ns simulation.

Supplementary Movie 3: Molecular dynamics simulation of PCBP1–*ILPR*-i-motif complex for 2500 ns. The video depicts the structural evolution of structure 1 under set (b) partially protonated condition. *ILPR*-i-motif backbone (silver), unprotonated cytosines (red), protonated cytosines (violet), KH1 domain (cyan), KH2 domain (light green), KH3 domain (yellow), interconnecting domains (violet). Progressive unfolding events, base-pair disruptions, or stabilization features can be observed over the course of the 2500 ns simulation.

Supplementary Movie 4: Molecular dynamics simulation of PCBP1–*ILPR*-i-motif complex for 2500 ns. The video depicts the structural evolution of structure 1 under set (c) fully protonated condition. *ILPR*-i-motif backbone (silver), unprotonated cytosines (red), protonated cytosines (violet), KH1 domain (cyan), KH2 domain (light green), KH3 domain (yellow), interconnecting domains (violet). Progressive unfolding events, base-pair disruptions, or stabilization features can be observed over the course of the 2500 ns simulation.
