# Supplementary material for: Mechanistic insights into PCBP1-driven unfolding of selected i-motif DNA at G1/S checkpoint
Source: Nat Commun. 2026 Feb 2;17:1149. doi: 10.1038/s41467-026-68822-5 (PMC12865031; doi:10.1038/s41467-026-68822-5)
Supplement: Supplementary file 13 — Reporting Summary [file 41467_2026_68822_MOESM13_ESM.pdf]

nature portfolio

Corresponding author(s): Professor Nasim Sabouri

Last updated by author(s): Jan 2, 2026

Reporting Summary

Nature Portfolio wishes to improve the reproducibility of the work that we publish. This form provides structure for consistency and transparency in reporting. For further information on Nature Portfolio policies, see our Editorial Policies and the Editorial Policy Checklist.

Statistics

For all statistical analyses, confirm that the following items are present in the figure legend, table legend, main text, or Methods section.

n/a	Confirmed	
☐	☒	The exact sample size (*n*) for each experimental group/condition, given as a discrete number and unit of measurement
☐	☒	A statement on whether measurements were taken from distinct samples or whether the same sample was measured repeatedly
☐	☒	The statistical test(s) used AND whether they are one- or two-sided<br>*Only common tests should be described solely by name; describe more complex techniques in the Methods section.*
☒	☐	A description of all covariates tested
☒	☐	A description of any assumptions or corrections, such as tests of normality and adjustment for multiple comparisons
☐	☒	A full description of the statistical parameters including central tendency (e.g. means) or other basic estimates (e.g. regression coefficient) AND variation (e.g. standard deviation) or associated estimates of uncertainty (e.g. confidence intervals)
☐	☒	For null hypothesis testing, the test statistic (e.g. *F*, *t*, *r*) with confidence intervals, effect sizes, degrees of freedom and *P* value noted<br>*Give P values as exact values whenever suitable.*
☒	☐	For Bayesian analysis, information on the choice of priors and Markov chain Monte Carlo settings
☒	☐	For hierarchical and complex designs, identification of the appropriate level for tests and full reporting of outcomes
☒	☐	Estimates of effect sizes (e.g. Cohen's *d*, Pearson's *r*), indicating how they were calculated

Our web collection on statistics for biologists contains articles on many of the points above.

Software and code

Policy information about availability of computer code

Data collection	1. Amersham Typhoon control software for image acquisition of the gels used in EMSA, i-motif-destabilization trapping assay, bromine footprinting, and high-resolution-primer extension assay.<br>2. Jasco J1700 CD spectral manager and spectral analyses for CD experiments.<br>3. NanoTemper Technologies MO.Control (v1.6.1) for MST experiments.<br>4. CFX96TM realtime system (BioRad) for ChIP-qPCR and thermal-shift assays.<br>5. Malvern Microcal PEAQ-ITC software for ITC experiments.<br>6. ChemiDoc MP system with Image Lab™ for image acquisition in western blot and anti-BrdU dot blot assay.<br>7. BD CSampler Plus Software version:1.0.34.1 for flow cytometry data acquisition.<br>8. Topspin 4.2.0 (Bruker) for 1D NMR spectral acquisition.<br>9. Amber20 package for molecular dynamics and simulation.<br>10. Alphafold, RoseTTAFold, and RoseTTAFoldNA for docking.
Data analysis	1. OriginPro 2020 for plotting and statistical analyses.<br>2. Microsoft excel used for basic calculations like SD, mean.<br>3. ImageJ for densitometric analyses of bands and dots in the gel images and dot blots, respectively.<br>4. Malvern Microcal PEAQ-ITC software for ITC experimental parameters calculation.<br>5. FCS express for flow cytometry data analyses.<br>6. RStudio 2024.09.0+375 and R 4.4.0 for creating Sankey plot and proteomic data analyses.

For manuscripts utilizing custom algorithms or software that are central to the research but not yet described in published literature, software must be made available to editors and reviewers. We strongly encourage code deposition in a community repository (e.g. GitHub). See the Nature Portfolio guidelines for submitting code & software for further information.

Data

Policy information about availability of data

All manuscripts must include a data availability statement. This statement should provide the following information, where applicable:

- Accession codes, unique identifiers, or web links for publicly available datasets
- A description of any restrictions on data availability
- For clinical datasets or third party data, please ensure that the statement adheres to our policy

The data that support this study are present in the manuscript and supplementary information. Source data are provided with this paper, which includes the raw data corresponding to all the graphs and plots in the main and supplementary figures; raw bruker datasets for NMR fids, and flow cytometry raw .fcs files. In ChIP-qPCR experiments, raw Ct values for Input, mock (IgG), and IP of all 3 biological replicates (having 3 technical replicates each) and related calculations are provided separately in Supplementary datasets 1-6, under a zipped folder, called 'additional data-ChIP-raw Ct values'. MD simulation trajectories are uploaded in zenodo and publicly available at https://doi.org/10.5281/zenodo.17737326.

Research involving human participants, their data, or biological material

Policy information about studies with human participants or human data. See also policy information about sex, gender (identity/presentation), and sexual orientation and race, ethnicity and racism.

Reporting on sex and gender	*Use the terms sex (biological attribute) and gender (shaped by social and cultural circumstances) carefully in order to avoid confusing both terms. Indicate if findings apply to only one sex or gender; describe whether sex and gender were considered in study design; whether sex and/or gender was determined based on self-reporting or assigned and methods used.<br>Provide in the source data disaggregated sex and gender data, where this information has been collected, and if consent has been obtained for sharing of individual-level data; provide overall numbers in this Reporting Summary. Please state if this information has not been collected.<br>Report sex- and gender-based analyses where performed, justify reasons for lack of sex- and gender-based analysis.*
Reporting on race, ethnicity, or other socially relevant groupings	*Please specify the socially constructed or socially relevant categorization variable(s) used in your manuscript and explain why they were used. Please note that such variables should not be used as proxies for other socially constructed/relevant variables (for example, race or ethnicity should not be used as a proxy for socioeconomic status).<br>Provide clear definitions of the relevant terms used, how they were provided (by the participants/respondents, the researchers, or third parties), and the method(s) used to classify people into the different categories (e.g. self-report, census or administrative data, social media data, etc.)<br>Please provide details about how you controlled for confounding variables in your analyses.*
Population characteristics	*Describe the covariate-relevant population characteristics of the human research participants (e.g. age, genotypic information, past and current diagnosis and treatment categories). If you filled out the behavioural & social sciences study design questions and have nothing to add here, write "See above."*
Recruitment	*Describe how participants were recruited. Outline any potential self-selection bias or other biases that may be present and how these are likely to impact results.*
Ethics oversight	*Identify the organization(s) that approved the study protocol.*

Note that full information on the approval of the study protocol must also be provided in the manuscript.

Field-specific reporting

Please select the one below that is the best fit for your research. If you are not sure, read the appropriate sections before making your selection.

☒ Life sciences ☐ Behavioural & social sciences ☐ Ecological, evolutionary & environmental sciences

For a reference copy of the document with all sections, see nature.com/documents/nr-reporting-summary-flat.pdf

Life sciences study design

All studies must disclose on these points even when the disclosure is negative.

Sample size	Sample sizes were based on established practices in biochemical and molecular biology standard practices with assay reproducibility. For quantified experiments, sample sizes of n = 3 biological replicates were used to ensure reproducibility and consistency of the observed effects. For ChIP-qPCR experiments, biological replicates (n = 3) were used, with each replicate representing an independently prepared chromatin sample. Within each biological replicate, qPCR was performed in 3 technical triplicates to ensure consistency.
Data exclusions	In ITC experiments, the first injection was excluded from analysis due to known artefacts caused by syringe backlash and diffusion effects (not true binding). This exclusion is standard and was automatically applied by the Malvern Microcal PEAQ-ITC software. All other data points were included. Except ITC, in all other experiments, no data points have been excluded for analyses.
Replication	All key experiments were independently replicated at least 3 times, yielding consistent results. Representative data are shown in the figures,

Replication	and quantified results are based on mean ± standard deviation of independent replicates.
Randomization	Randomisation was not applicable, as the study did not involve treatment groups, animals, or patient cohorts. Our study is based on in vitro biochemical or molecular biology studies and cell-based functional assays.
Blinding	Blinding was not applicable as the study involved in vitro molecular biology and biophysical assays with objective, instrument-based readouts. No treatment groups or subjective scoring were used.

Reporting for specific materials, systems and methods

We require information from authors about some types of materials, experimental systems and methods used in many studies. Here, indicate whether each material, system or method listed is relevant to your study. If you are not sure if a list item applies to your research, read the appropriate section before selecting a response.

Materials & experimental systems

n/a	Involved in the study	
☐	☒	Antibodies
☐	☒	Eukaryotic cell lines
☒	☐	Palaeontology and archaeology
☒	☐	Animals and other organisms
☒	☐	Clinical data
☒	☐	Dual use research of concern
☒	☐	Plants

Methods

n/a	Involved in the study	
☒	☐	ChIP-seq
☐	☒	Flow cytometry
☒	☐	MRI-based neuroimaging

Antibodies

Antibodies used	1. Anti-PCBP1 antibody: Anti-PCBP1 antibody; clone number: EPR11049(B), catalog number: ab168377, IgG isotype, Clonality: monoclonal, Host: rabbit; make: Abcam; dilution: 1:500 in ChIP, 1:1000 in western blot; Lot number: 1050664-5<br>2. iMab antibody: Anti-i-motif DNA [iMab], scFv fragment (3xFLAG + His), ScFv; Catalog number: Ab01462-30.135, Absolute antibody Amount: 2 micrograms<br>3. BG4 antibody: prepared in house and details of purification provided in our prior publication: Deiana et al. A new G-quadruplex-specific photosensitizer inducing genome instability in cancer cells by triggering oxidative DNA damage and impeding replication fork progression, Nucleic Acids Research, Volume 51, Issue 12, 7 July 2023, Pages 6264–6285; amount: 500 ng<br>4. DYKDDDDK Tag (D6W5B) Rabbit mAb anti-FLAG antibody, catalog number: 14793S, make: Cell Signalling, Clonality: Monoclonal; IgG isotype; amount: 8 micrograms); Lot number: 7<br>5. Rabbit Phospho-Histone H2A.X (Ser139) Antibody, Catalog number: 2577S, make: Cell Signalling, clonality: polyclonal; IgG isotype; dilution: 1:1000. Lot number: 14<br>6. Anti-BrdU antibody [BU1/75 (ICR1)], catalog number: ab6326, make: Abcam, Clonality: monoclonal, subtype: IgG2a; dilution: 1:1000<br>7. Goat Anti-Rat IgG Peroxidase Conjugate, DC01L-200UG, Merck, IgG isotype (1:5000)<br>8. Anti-beta Actin antibody [mAbcam 8224] - Loading Control, catalog number: ab8224, make: Abcam, IgG1 isotype, monoclonal (1:5000). Lot number: 1051636-14<br>9. Goat anti-Rabbit IgG (H+L) Secondary Antibody, HRP, Thermo-fisher scientific, 31460, polyclonal (1:5000).
Validation	Primary and secondary antibodies are used according to the commercial suppliers protocols.<br>BG4 Antibody specificity was previously published and validated by Biffi et al (10.1038/nchem.1548) and also validated by us in Deiana et al (10.1093/nar/gkad365).<br>For some antibodies, like iMab, anti-BrDU, Goat Anti-Rat IgG Peroxidase Conjugate, and Goat anti-Rabbit IgG (H+L) Secondary Antibody, we used antibodies from different lot numbers and they are validated in the lab. The result is consistent across different lot numbers.

Eukaryotic cell lines

Policy information about cell lines and Sex and Gender in Research

Cell line source(s)	HeLa (ATCC), female, cervical carcinoma
Authentication	Good lab practice was used to set the basic benchmark of cell verification using low-passage and morphology check.
Mycoplasma contamination	We confirm that the cells used in this manuscript have been tested negative for mycoplasma contamination.
Commonly misidentified lines (See ICLAC register)	not applicable

Plants

Seed stocks

Report on the source of all seed stocks or other plant material used. If applicable, state the seed stock centre and catalogue number. If plant specimens were collected from the field, describe the collection location, date and sampling procedures.

Novel plant genotypes

Describe the methods by which all novel plant genotypes were produced. This includes those generated by transgenic approaches, gene editing, chemical/radiation-based mutagenesis and hybridization. For transgenic lines, describe the transformation method, the number of independent lines analyzed and the generation upon which experiments were performed. For gene-edited lines, describe the editor used, the endogenous sequence targeted for editing, the targeting guide RNA sequence (if applicable) and how the editor was applied.

Authentication

Describe any authentication procedures for each seed stock used or novel genotype generated. Describe any experiments used to assess the effect of a mutation and, where applicable, how potential secondary effects (e.g. second site T-DNA insertions, mosiacism, off-target gene editing) were examined.

Flow Cytometry

Plots

Confirm that:

☒ The axis labels state the marker and fluorochrome used (e.g. CD4-FITC).

☒ The axis scales are clearly visible. Include numbers along axes only for bottom left plot of group (a 'group' is an analysis of identical markers).

☐ All plots are contour plots with outliers or pseudocolor plots.

☒ A numerical value for number of cells or percentage (with statistics) is provided.

Methodology

Sample preparation

HeLa cells were sub-cultured at a density of 1,000,000 cells per well. Following treatment with either double-thymidine block or PCBP1-knockdown. Cells were harvested using trypsinization and subsequently washed with cold 1× PBS. The cells were then fixed with chilled 70% ethanol and stored at −20 °C overnight. After fixation, the cells were centrifuged at 850 × g for 15 minutes at 4 °C. The resulting pellets were resuspended in 500 µL of FxCycle PI/RNase staining solution (Invitrogen) and incubated in the dark at 4 °C with gentle rocking for 4 hours. DNA content was analyzed using flow cytometry.

Instrument

BD Accuri C6 Plus

Software

FCS express and BD CSampler Plus Software version:1.0.34.1.

Cell population abundance

The abundance of each cell population was determined as a percentage of singlet cells after gating. Fluorescent marker-positive cells (Phycoerythrin) were quantified relative to total events acquired.

Gating strategy

Cells were first gated to exclude debris using FSC-A vs SSC-A, followed by doublet discrimination using FSC-H vs FSC-A. Fluorescent marker (Phycoerythrin)-positive populations were identified using appropriate unstained controls. The abundance of each population was calculated as a percentage of total singlet cells.

☐ Tick this box to confirm that a figure exemplifying the gating strategy is provided in the Supplementary Information.
