# Supplementary figures and images for: Mechanistic insights into PCBP1-driven unfolding of selected i-motif DNA at G1/S checkpoint
Source: Nat Commun. 2026 Feb 2;17:1149. doi: 10.1038/s41467-026-68822-5 (PMC12865031; doi:10.1038/s41467-026-68822-5)

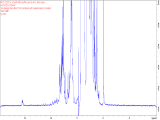

Supplement: Supplementary file 15 — Source data [file 41467_2026_68822_MOESM15_ESM.zip › Source data/Source data_Main figures/Source data_NMR/Source data_Fig. 3A/Myc-C20T+PCBP1/1/pdata/1/thumb.png]

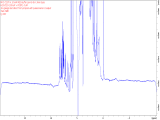

Supplement: Supplementary file 15 — Source data [file 41467_2026_68822_MOESM15_ESM.zip › Source data/Source data_Main figures/Source data_NMR/Source data_Fig. 3A/Myc-C20T+PCBP1/4/pdata/1/thumb.png]

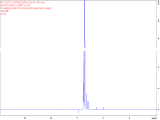

Supplement: Supplementary file 15 — Source data [file 41467_2026_68822_MOESM15_ESM.zip › Source data/Source data_Main figures/Source data_NMR/Source data_Fig. 3A/Myc-C20T+PCBP1/5/pdata/1/thumb.png]

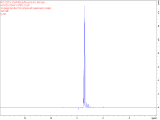

Supplement: Supplementary file 15 — Source data [file 41467_2026_68822_MOESM15_ESM.zip › Source data/Source data_Main figures/Source data_NMR/Source data_Fig. 3A/Myc-C20T+PCBP1/6/pdata/1/thumb.png]

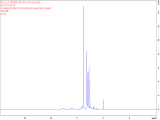

Supplement: Supplementary file 15 — Source data [file 41467_2026_68822_MOESM15_ESM.zip › Source data/Source data_Main figures/Source data_NMR/Source data_Fig. 4E/BCL2+PCBP1/1/pdata/1/thumb.png]

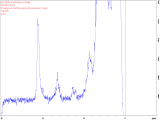

Supplement: Supplementary file 15 — Source data [file 41467_2026_68822_MOESM15_ESM.zip › Source data/Source data_Main figures/Source data_NMR/Source data_Fig. 4E/BCL2+PCBP1/49/pdata/1/thumb.png]

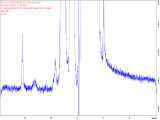

Supplement: Supplementary file 15 — Source data [file 41467_2026_68822_MOESM15_ESM.zip › Source data/Source data_Main figures/Source data_NMR/Source data_Fig. 4E/BCL2+PCBP1/7/pdata/1/thumb.png]
